# Higher-valency pneumococcal conjugate vaccines in older adults, taking into account indirect effects from childhood vaccination: a cost-effectiveness study for the Netherlands

**DOI:** 10.1186/s12916-024-03277-3

**Published:** 2024-02-16

**Authors:** Pieter T. de Boer, Cornelis H. van Werkhoven, Albert Jan van Hoek, Mirjam J. Knol, Elisabeth A. M. Sanders, Jacco Wallinga, Hester E. de Melker, Anneke Steens

**Affiliations:** 1 Center for Infectious Disease Control, National Institute for Public Health and the Environment (RIVM), Bilthoven, the Netherlands; 2 Julius Center for Health Sciences and Primary Care, University Medical Center Utrecht, Utrecht University, Utrecht, the Netherlands; 3 Department of Pediatric Immunology and Infectious Diseases, University Medical Center Utrecht, Utrecht, the Netherlands; 4 Department of Biomedical Datasciences, Leiden University Medical Center, Leiden, the Netherlands

**Keywords:** Pneumococcal, Vaccination, Cost-effectiveness, Economic evaluation, Serotype replacement

## Abstract

**Background:**

New 15- and 20-valent pneumococcal vaccines (PCV15, PCV20) are available for both children and adults, while PCV21 for adults is in development. However, their cost-effectiveness for older adults, taking into account indirect protection and serotype replacement from a switch to PCV15 and PCV20 in childhood vaccination, remains unexamined.

**Methods:**

We used a static model for the Netherlands to assess the cost-effectiveness of different strategies with 23-valent pneumococcal polysaccharide vaccine (PPV23), PCV15, PCV20, and PCV21 for a 65-year-old cohort from a societal perspective, over a 15-year time horizon. Childhood vaccination was varied from PCV10 to PCV13, PCV15, and PCV20. Indirect protection was assumed to reduce the incidence of vaccine serotypes in older adults by 80% (except for serotype 3, no effect), completely offset by an increase in non-vaccine serotype incidence due to serotype replacement.

**Results:**

Indirect effects from childhood vaccination reduced the cost-effectiveness of vaccination of older adults, depending on the serotype overlap between the vaccines. With PCV10, PCV13, or PCV15 in children, PCV20 was more effective and less costly for older adults than PPV23 and PCV15. PCV20 costs approximately €10,000 per quality-adjusted life year (QALY) gained compared to no pneumococcal vaccination, which falls below the conventional Dutch €20,000/QALY gained threshold. However, with PCV20 in children, PCV20 was no longer considered cost-effective for older adults, costing €22,550/QALY gained. As indirect effects progressed over time, the cost-effectiveness of PCV20 for older adults further diminished for newly vaccinated cohorts. PPV23 was more cost-effective than PCV20 for cohorts vaccinated 3 years after the switch to PCV20 in children. PCV21 offered the most QALY gains, and its cost-effectiveness was minimally affected by indirect effects due to its coverage of 11 different serotypes compared to PCV20.

**Conclusions:**

For long-term cost-effectiveness in the Netherlands, the pneumococcal vaccine for older adults should either include invasive serotypes not covered by childhood vaccination or become more affordable than its current pricing for individual use.

**Supplementary Information:**

The online version contains supplementary material available at 10.1186/s12916-024-03277-3.

## Background

The *Streptococcus pneumoniae* bacterium causes significant morbidity, mortality, and economic burden in older adults [1]. Its most severe clinical picture is invasive pneumococcal disease (IPD), with a case fatality rate of 10–30% [2]. Additionally, *S. pneumoniae* is responsible for 9–30% of community acquired pneumonia (CAP) cases [3], a leading cause of deaths among older adults. More than 100 pneumococcal serotypes exist. Two vaccines have been used in older adults: a 23-valent pneumococcal polysaccharide vaccine (PPV23) and a 13-valent pneumococcal conjugate vaccine (PCV13). PPV23 contains more serotypes but provides less effective and shorter-lasting protection against vaccine-type serotypes due to the absence of memory B cell activation [4].

Childhood vaccination programs with PCVs have significantly altered the serotype distribution of IPD cases among older adults. In the Netherlands, no programmatic pneumococcal vaccination for older adults existed before 2020. Indirect protection from childhood vaccination with PCV7 since 2006 and PCV10 since 2011, both with over 90% vaccine uptake [5], reduced the incidence of IPD from PCV10 serotypes by 96% among ≥ 60-year-olds between 2007 and 2019 [6]. However, the burden of pneumococcal disease in older adults remains substantial due to an increase of non-PCV10 serotypes through serotype replacement. Indirect effects from childhood vaccination have also reduced the cost-effectiveness of pneumococcal vaccination for older adults, particularly for PCV13 [7, 8], which saw a reduction in IPD incidence for 10 of its 13 serotypes. The cost-effectiveness of PPV23 was not substantially affected, as it covers additional serotypes. As a result, the Netherlands opted for the use of PPV23 for its program for older adults aged 60–79 years, starting in 2020. To prevent suboptimal protection due to waning immunity, revaccination is recommended every 5 years. Also, many other high-income countries recommend PPV23 for older adults, either alone or 1 year after an initial dose with PCV13.

Recognizing the limitations of PPV23 regarding its immunogenicity, higher-valency PCVs have emerged. In 2022, the European Medicine Agency licensed PCV15 for adults and children and PCV20 for adults with anticipated licensing for children. These vaccines expand on PCV13, adding two and seven serotypes, respectively. Additionally, PCV21, specifically formulated for adults, has entered phase 3 trials. PCV21 excludes nine PCV10 serotypes and introduces 11 new serotypes compared to PCV20, based on the serotypes most prevalent in adult IPD cases. Previous studies, except for one US study [9], suggest that PCV20 is cost-effective or cost-saving for older adults compared to PPV23, PCV13, and PCV15 [10–22], while PCV21 was estimated cost-saving compared to PCV20 [23] (see Additional file 1: Table S1 for details). However, half of these studies did not account for the indirect protection resulting from a possible switch to PCV15 or PCV20 in the childhood vaccination program [11, 13, 15–19, 21]. Two studies considering serotype replacement did not address the cost-effectiveness of PCV21 [20, 22].

We evaluated the cost-effectiveness of different vaccination strategies with PPV23, PCV15, PCV20, and PCV21 in the Netherlands, taking into account indirect protection and serotype replacement from a switch to higher-valency PCVs in childhood vaccination. While focusing on the Netherlands, our findings hold wider policy implications for all countries considering higher-valency PCVs in older adults.

## Methods

### Analysis framework

We updated a previously published static multi-cohort model [7] to compare costs and quality-adjusted life year (QALY) losses of various pneumococcal vaccination strategies for older adults in the Netherlands. The primary analysis focused on a 65-year-old cohort over a 15-year time horizon. We also explored alternative vaccination ages (60, 70, 75, 80, and 85 years). The following strategies were evaluated:

1. No pneumococcal vaccination
2. Three doses of PPV23 (3xPPV23, years 0, 5, and 10)
3. PCV15 (year 0)
4. PCV20 (year 0)
5. PCV15 + 3xPPV23 (year 0 + years 1, 6, and 11)
6. PCV20 + 3xPPV23 (year 0 + years 1, 6, and 11)
7. PCV21 (year 0)

Strategy 2 aligns with the current Dutch PPV23 program.

The cost-effectiveness of vaccination of older adults was assessed with the continuation of PCV10 in childhood vaccination, or with a switch to PCV13, PCV15, or PCV20 at year 0, resulting in indirect effects on older adults. Notably, PCV21 is not considered for childhood vaccination, as its serotypes have been selected based on the pneumococcal serotype epidemiology in older adults. Moreover, PCV21 is unadjuvanted and, therefore, potentially less immunogenic in children < 2 years of age compared to other PCVs [24]. Indirect effects from childhood vaccination were included by adjusting the incidence of infection in older adults with a correction factor (see the “Indirect effects from childhood vaccination” section). As our analysis is centered on informing decisions regarding the vaccination of older adults, we did not include additional vaccination costs associated with a switch to higher-valency PCVs in childhood vaccination. Furthermore, the analysis does not account for the direct or indirect impact of these higher-valency vaccines on the disease burden in children themselves or in younger adults.

### Model and input data

A comprehensive model description and the parameterization details are available in the Supplementary Methods of Additional file 1. Briefly, our model tracked single year of age cohorts aged ≥ 60 years in annual time steps, in which they could develop IPD or hospitalized non-invasive pneumococcal pneumonia (NIPP). Pneumococcal infections requiring primary care were not included, given the uncertainty regarding whether pneumococcal vaccination provides significant protection against this outcome [25]. To avoid the influence of COVID-19 measures on pneumococcal epidemiology [26], we utilized pre-2020 epidemiological data. As the programmatic pneumococcal vaccination for older adults started in the Netherlands in 2020, this concerned data from an unvaccinated population. Consequently, there is no need for back calculation to determine the burden of pneumococcal disease in an unvaccinated population. The average IPD incidence by age during the years 2017–2019 was derived from Dutch national surveillance data. The NIPP incidence by age was estimated from the average all-cause CAP hospitalization incidence during 2012–2014, assuming that 22.1% of these cases were caused by *S. pneumoniae* [27]. NIPP incidence was adjusted to account for overlap with IPD cases [28] and indexed to 2017–2019 using the IPD time trend. IPD and NIPP cases were categorized by serotype based on data of 2019 from Dutch IPD cases aged ≥ 60-year-olds. Age-specific 30-day case fatality rates for IPD and NIPP were used to estimate pneumococcal-related deaths [29, 30]. Cohort sizes and aging of cohorts were informed by data from Statistics Netherlands [31, 32].

The impact of vaccination was modeled as a reduction in the incidence of vaccine serotypes, involving the vaccine uptake and vaccine-type specific vaccine effectiveness (VE). A vaccination coverage of 70% reflected the current uptake among Dutch older adults [33, 34]. The VE at time of vaccination decreased with increasing age. At age 65 years, the vaccine-type specific VE for PPV23 was estimated at 54% against IPD and 27% against NIPP, using data from multiple observational studies [35–37]. A revaccination with PPV23 was assumed to have the same VE as a first dose would have had at that age. For PCV, a vaccine-type specific VE of 81% against IPD and 54% against NIPP for vaccination at age 65 years was derived from age-specific VE estimates in a post hoc analysis of Dutch trial data for PCV13 [38]. This VE was adjusted downwards to account for the relatively healthy trial population. The vaccine-type specific VE for PCV13 was extrapolated to PCV15, PCV20, and PCV21 serotypes, and it was also applied to non-vaccine serotype 6C, recognizing evidence on cross-protection via serotype 6A [39]. The decrease of VE over time due to waning immunity was modeled similarly to previous assumptions [7]. For PPV23, the VE remained constant for 2 years and then linearly decreased to 0% at 5 years post-vaccination. For PCV, the VE remained constant for 4 years, and then linearly declined to 0% at 15 years post-vaccination. For combined strategies with PCV and PPV23, the VE of the vaccine with the higher VE was utilized at each time step; no additional efficacy against serotypes covered by both vaccines was assumed.

### Indirect effects from childhood vaccination

With continued use of PCV10 in childhood vaccination, we assumed a steady pneumococcal disease incidence and serotype distribution in older adults. With the implementation PCV13, PCV15, or PCV20 in childhood vaccination, we based the time courses of indirect protection and serotype replacement on observations from the multi-country studies PSERENADE and SpIDnet after the introduction of PCV10 and PCV13 childhood programs [40, 41]. The PSERENADE data shows an over 75% decrease in the incidence of IPD from PCV10-included serotypes in individuals aged ≥ 65 years at 8 years after the initiation of the childhood PCV program. Subsequently, there was an observed increase in IPD incidence from non-vaccine serotypes, attributed to serotype replacement. The net impact on IPD incidence varied across countries, with some experiencing no change, while others saw up to a 50% decline. In the Netherlands, despite an over 90% reduction in IPD incidence from PCV10-included serotypes, there was no significant net change in IPD incidence among individuals aged ≥ 60 during the period 2008–2019 (Additional file 1: Figure S2). Taking these observations into account, we used the following assumptions for our main analysis:

1. Due to indirect protection, the incidence of childhood vaccine serotypes added to PCV10 decreased by 80% in older adults. This decrease occurred linearly, starting 1 year post-implementation and completing 8 years post-implementation. No indirect protection was assumed for serotype 3 [41]. Cross-protection to serotype 6C was included.
2. Due to serotype replacement, the incidence of non-childhood vaccine serotypes increased in older adults until the incidence of all serotypes reached the pre-indirect effects level (i.e., 100% replacement). This increase occurred linearly, starting 3 years post-implementation and completing 8 years post-implementation. The relative contribution of different non-vaccine serotypes remained constant during the increase.

**Fig. 1:**
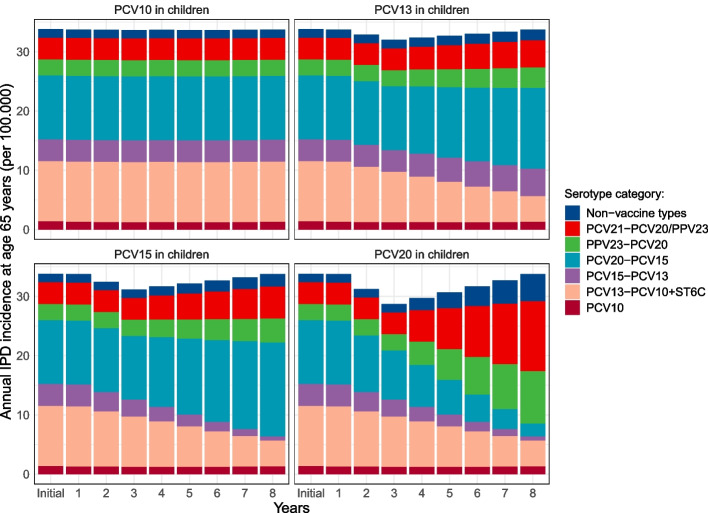
Projected incidence and serotype distribution per 100,000 persons at the age of 65 years in the Netherlands over time in case of the continuation of PCV10 (no indirect effects) or after applying indirect effects from a switch to PCV13, PCV15, or PCV20 in childhood vaccination. Indirect effects encompass 80% indirect protection for serotypes added to PCV10 (starting 1 year after the switch and completed 8 years after the switch), with pneumococcal incidence levels returning to pre-indirect effects level due to serotype replacement (starting 3 years after the switch and completed 8 years after the switch). After 8 years, the incidence and serotype distribution remained stable for the rest of the time horizon. The split into serotype categories is based on Dutch serotype distribution data from 2019, when no programmatic pneumococcal vaccination occurred in the Netherlands. Serotype per category PCV10: 4, 6B, 9V, 14, 18C, 19F, 23F, 1, 5, 7F; PCV13-PCV10 + ST6C: 3, 6A, 19A, 6C (cross-protection via 6A), PCV15-PCV13: 22F, 33F, PCV20-PCV15: 8, 10A, 11A, 12F, 15B; PPV23-PCV20: 2, 9N, 17F, 20; PCV21-PCV20/PPV23: 15A, 15C, 16F, 23A, 23B, 24F, 31, 35B; PCV, pneumococcal conjugate vaccine; PPV, pneumococcal polysaccharide vaccine; IPD, invasive pneumococcal disease

Figure 1 illustrates the change in incidence and serotype distribution of IPD at the age of 65 years over time. The new steady state reached 8 years post-switch was maintained for the remaining time horizon. Recognizing the uncertainty associated with these assumptions, we performed a sensitivity analyses by altering the magnitudes of indirect effects. Considering modeling suggestions that an increase in vaccine valency could diminish indirect effects [42], we explored a scenario incorporating 40% indirect protection. Regarding serotype replacement, we investigated a scenario in which 50% of the reduction in pneumococcal disease burden was replaced.

### Economic evaluation

The societal perspective was taken, following Dutch guidelines [43]. We used IPD- and NIPP-related hospitalization costs, patient expenses, and productivity losses for the Netherlands [30, 44–46], adjusted to the 2021 price year [47] (Additional file 1: Table S4). Vaccine administration costs at the general practitioner in 2021 were €21 per dose [48]. Vaccine prices per dose were acquired from a Dutch pricelist for individual usage: PPV23 at €25.94, PCV15 at €74.73, and PCV20 at €82.17 [49]. In the absence of a price for the in-development PCV21, we assumed it to have the same price as PCV20. This approach ensures that any differences in costs between PCV20 and PCV21 can be attributed solely to the prevented economic burden. This does not imply a prediction that the eventual price of PCV21 will be the same as PCV20. QALYs lost due to either IPD or NIPP was 0.0709, based on utility loss during the acute phase and first month of hospitalized non-fatal CAP cases [46]. QALYs lost from premature death were age-specific, using the life expectancy adjusted for general population utilities (Additional file 1: Table S5 and Figure S5).

### Cost-effectiveness

Costs and QALY losses for each strategy were accumulated over the time horizon, with annual discount rates of 4% for costs and 1.5% for QALYs [43]. Incremental cost-effectiveness ratios (ICERs) were calculated, with strongly dominated (i.e., presence of a more effective and less costly alternative) and extendedly dominated strategies (presence of a more effective alternative with lower ICER) being removed from the comparison. Probabilistic sensitivity analyses involved 1000 Monte Carlo simulations, with all parameter values drawn from their distributions. The 2.5% and 97.5% percentiles informed 95% confidence intervals (95% CI) of estimated case reductions. Uncertainty of cost-effectiveness outcomes was displayed using cost-effectiveness acceptability frontiers, illustrating the preferred strategy at different willingness-to-pay (WTP) thresholds. A common WTP threshold for preventive interventions in the Netherlands is €20,000/QALY gained [50]. One-way sensitivity analyses assessed the impact of assumptions or alternative sources on the cost-effectiveness outcomes (Additional file 1: Table S6) [51–53].

## Results

### Clinical impact

**Fig. 2:**
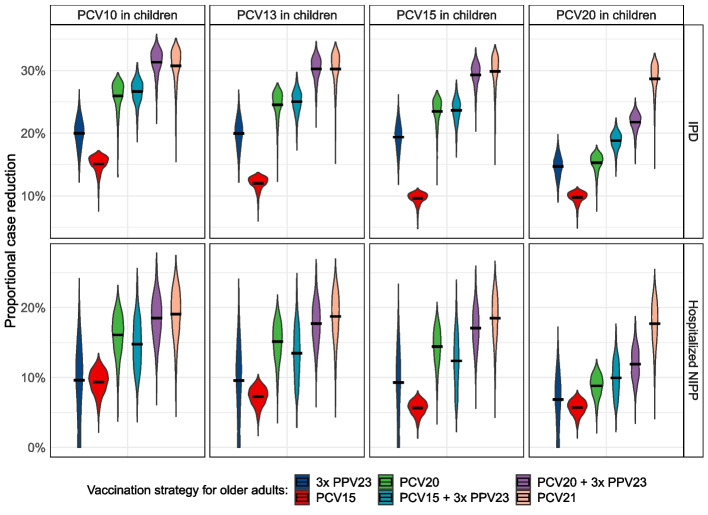
Proportional reductions in IPD cases (top) and hospitalized NIPP cases (bottom) for different pneumococcal vaccination strategies for older adults (colors) compared to no pneumococcal vaccination, while varying the childhood vaccination strategy (columns). Results are based on a 65-year-old cohort, vaccinated at 70% vaccination coverage and followed over 15-year period. The main analysis estimate is depicted by the black line, while the distributions are based on 1000 Monte Carlo simulations in a probabilistic sensitivity analysis. 3xPPV23: PPV23 administered at years 0, 5, and 10; PCV15 + 3xPPV23: PCV15 administered at year 0 and PPV23 at years 1, 6, and 11; PCV20 + 3xPPV23: PCV20 administered at year 0 and PPV23 at years 1, 6, and 11. IPD, invasive pneumococcal disease; NIPP, non-invasive pneumococcal pneumonia; PCV, pneumococcal conjugate vaccine; PPV, pneumococcal polysaccharide vaccine

Figure 2 shows the relative reductions in IPD cases and NIPP cases for different vaccination strategies in a 65-year-old cohort (228,209 individuals, 70% vaccination coverage) over a 15-year period compared to no vaccination, while varying the childhood vaccine. Absolute numbers of cases prevented are detailed in Additional file 1: Tables S7-S10. With continued use of PCV10 in children, the current Dutch strategy of 3xPPV23 was estimated to reduce the number of IPD cases by 20% (95% CI: 15–24%) and the number of NIPP hospitalizations by 10% (95% CI: 0–19%). This corresponds to 274 prevented IPD cases and 227 prevented NIPP hospitalizations and the avoidance of 69 deaths. PCV15 had a smaller impact, while PCV20 had a greater impact than 3xPPV23, resulting in a 26% (95% CI: 20–29%) reduction. PCV15 + 3xPPV23 had similar impact to PCV20, while PCV20 + 3xPPV23 had most impact, preventing 32% (95% CI: 26–34%) of IPD cases. PCV21, which is in development, prevented 31% (95% CI: 23–34%) of IPD cases. The impact of vaccination on NIPP was relatively lower compared to the impact on IPD, particularly for strategies involving PPV23 (Fig. 2, bottom left). PCV21 prevented most NIPP hospitalizations with a reduction of 19% (95% CI: 11–25%) compared to no vaccination.

Indirect effects from the use of higher-valency PCVs in childhood vaccination reduced the impact of pneumococcal vaccination in older adults, depending on the serotype overlap between the vaccines used for children and older adults (Fig. 2). A switch from PCV10 to PCV13 or to PCV15 in children reduced the impact of PCV15 in older adults substantially, but the impact of PPV23, PCV20, or PCV21 minorly. Use of PCV20 in children reduced the impact of both PCV20 and PPV23 in older adults, although the change in impact was larger for PCV20. In that scenario, PCV20 prevented 15% (95% CI: 12–17%) of IPD cases in the 65-years-old cohort, about equally high as the impact of 3xPPV23. Indirect effects from PCV20 in children did minimally affect the impact of PCV21 in older adults, preventing 29% (95% CI: 22–32%) of IPD cases in older adults.

### Cost-effectiveness

**Fig. 3:**
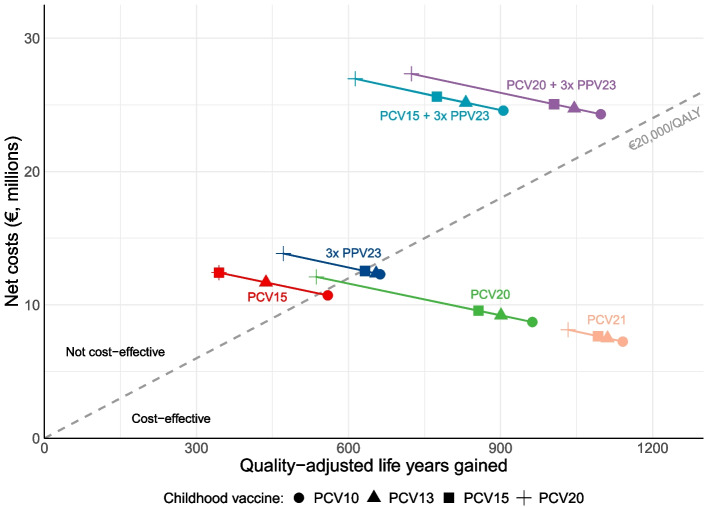
Discounted net costs and quality-adjusted life years gained of various pneumococcal vaccination strategies (indicated in colors) compared to no vaccination (*x* = 0, *y* = 0) in older adults, while varying the childhood vaccine (indicated by the symbols). Results are presented for a 65-year-old cohort with a vaccination coverage of 70%, followed for 15 years. For PCV21, we assumed that the vaccine price was equal to PCV20. 3xPPV23: PPV23 administered at years 0, 5, and 10; PCV15 + 3xPPV23: PCV15 administered at year 0 and PPV23 at years 1, 6, and 11; PCV20 + 3xPPV23: PCV20 administered at year 0 and PPV23 at years 1, 6, and 11. PCV, pneumococcal conjugate vaccine; PPV, pneumococcal polysaccharide vaccine; QALY, quality-adjusted life year

Figure 3 shows the discounted net costs and QALY gains for different vaccination strategies of a 65-year-old cohort over a 15-year period compared to no vaccination, while varying the childhood vaccine (details provided in Additional file 1: Tables S11-S14). With PCV10 in children, we found PCV20 to dominate 3xPPV23, PCV15, and PCV15 + 3xPPV23 in the 65-year-old cohort (Fig. 3, with higher QALY gains against lower costs). Compared to no vaccination, PCV20 resulted in a gain of 963 QALYs, from which 57% was attributed to prevented IPD cases and 43% to prevented NIPP cases. With net costs of €8.7 million, the ICER was estimated at €9051/QALY gained. Switching to PCV13 or PCV15 in childhood vaccination resulted in a slight increase of the ICER of PCV20 to €10,228/QALY gained and €11,173/QALY gained, respectively. However, the cost-effectiveness of PCV20 in older adults diminished substantially if children were vaccinated with PCV20; the ICER compared to no vaccination increased to €22,550/QALY gained, above the conventional Dutch threshold of €20,000/QALY gained. PCV20 + 3xPPV23 yielded a higher QALY gain in older adults than PCV20, though at an ICER of €81,193–€115,412/QALY gained compared to PCV20, depending on the childhood vaccination scenario. At equal vaccine prices, PCV21 dominated PCV20 for older adults. Indirect effects had minimal impact on the cost-effectiveness of PCV21; the ICER of PCV21 compared to no vaccination in older adults increased from €6352/QALY gained with PCV10 in children to €7876/QALY gained with PCV20 in children.

### Probabilistic sensitivity analysis

**Fig. 4:**
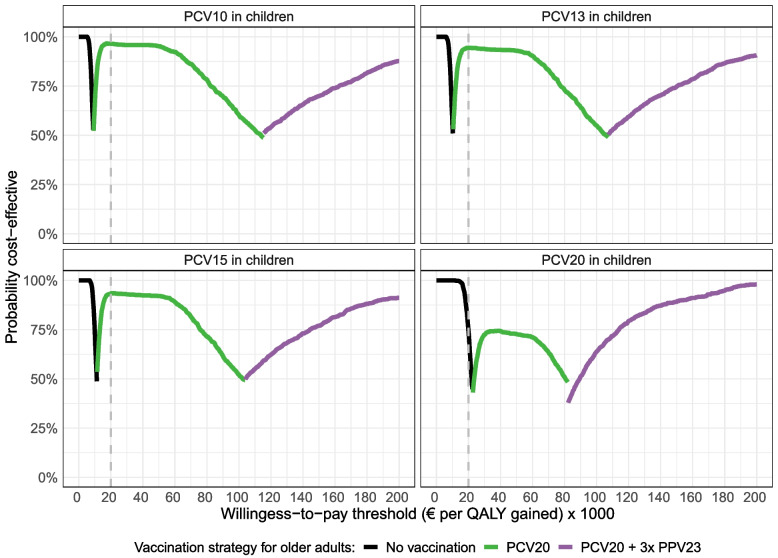
Cost-effectiveness acceptability frontiers of various pneumococcal strategies for older adults (different colored lines), while varying the childhood vaccine (different panels). The graph shows the probability of being the most cost-effective strategy across a range of WTP-thresholds, after using 1000 Monte Carlo simulations in the probabilistic sensitivity. Results are presented for a 65-year-old cohort with a vaccination coverage of 70%, followed for 15 years. Dominated strategies are removed. PCV21 is not included, as the vaccine is currently not available. PCV20 + 3xPPV23: PCV20 administered at year 0 and PPV23 at years 1, 6, and 11. CE, cost-effectiveness; PCV, pneumococcal conjugate vaccine; PPV, pneumococcal polysaccharide vaccine. The dashed vertical line indicates a commonly accepted WTP threshold

Focusing on current available vaccines for older adults, we found PCV20 to be the cost-effective strategy at a €20,000/QALY gained threshold (vertical dashed line) in 90% of the simulations if PCV10 was continued in children (Fig. 4, top left). However, with PCV20 in children, no pneumococcal vaccination was the cost-effective strategy for older adults at a €20,000/QALY gained threshold in 74% of the simulations (Fig. 4, bottom right).

### One-way sensitivity analyses

**Fig. 5:**
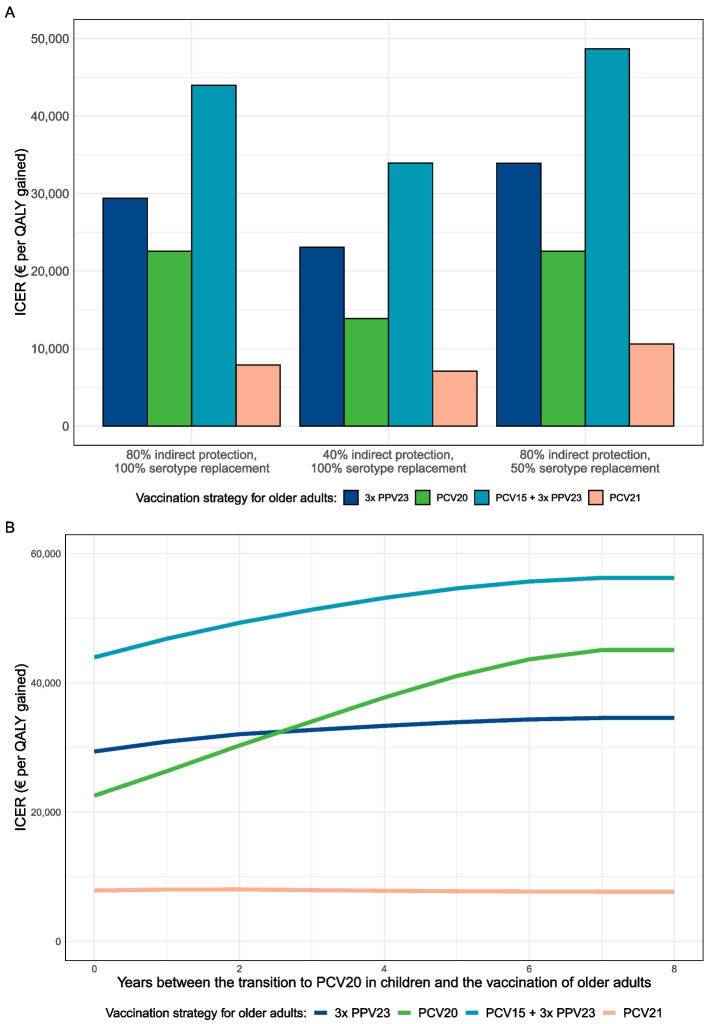
Sensitivity analysis of the incremental cost-effectiveness ratio (ICER) of various vaccination strategies for older adults when switching to PCV20 in childhood vaccination, compared to no vaccination. Panel **A** shows the impact of varying the magnitude of indirect protection or serotype replacement. The main analysis assumes 80% indirect protection with 100% serotype replacement (pneumococcal incidence levels returning to pre-indirect effects level). Panel **B** shows the impact of varying the time between the switch to PCV20 in childhood vaccination and the vaccination of older adults. The main analysis assumes older adults to be vaccinated in the year of switching (year 0 in the graph). Year 8 in the graph means that older adults are vaccinated 8 years after the implementation of PCV20 in childhood vaccination. Results are presented for a 65-year-old cohort with a 70% vaccination coverage, followed for 15 years. The vaccine price of PCV21 was assumed to be equal to PCV20. 3xPPV23: PPV23 administered at years 0, 5, and 10; PCV15 + 3xPPV23: PCV15 administered at year 0 and PPV23 at years 1, 6, and 11. ICER, incremental cost-effectiveness ratio; PCV, pneumococcal conjugate vaccine; PPV, pneumococcal polysaccharide vaccine; QALY, quality-adjusted life year

We found the results to be sensitive to the magnitude of indirect effects (Fig. 5A). For a scenario with 40% indirect protection from PCV20 in children (instead of 80%), the ICER of PCV20 compared to no vaccination for the 65-year-old cohort was €13,883/QALY gained (instead of €22,550/QALY gained). Reducing serotype replacement from PCV20 in children primarily diminished the cost-effectiveness of PCV21 for older adults. If only 50% of the IPD and NIPP cases prevented by indirect protection were replaced (instead of 100%), the ICER of PCV21 compared to no vaccination was €10,589/QALY gained (instead of €7876/QALY gained).

We tested other parameters and found that the results were most sensitive to the vaccine price, the VE and its waning rate, and the proportion of CAP caused by *S. pneumoniae* (Additional file 1: Figure S6). With PCV20 in childhood vaccination, PCV20 was cost-effective for a 65-year-old cohort at a €20,000/QALY threshold if the VE did not wane for at least 8 years or if its price was reduced by 13% (Additional file 1: Figure S6D). To achieve an ICER of €10,000/QALY compared to no vaccination, as was found for PCV20 in older adults with PCV10 in children, the price of PCV20 should be reduced by 50%. PPV23 could become cost-effective compared to PCV20 by using the upper bound of the VE of PPV23, the lower bound of the VE of PCV20, assuming no waning of PPV23 within 5 years, or reducing its price by 50%.

Varying the vaccination age, we found the cost-effectiveness results to be consistent within the range of 60–80 years. Vaccination at the age of 70 or 75 years resulted in the lowest ICERs (Additional file 1: Figures S7-S8 and Tables S15-S24). The cost-effectiveness of pneumococcal vaccination diminished substantially at the vaccination age of 85 years.

### Cost-effectiveness in future vaccinated cohorts

As indirect effects were modeled to progress gradually over time, its impact on the cost-effectiveness of pneumococcal vaccination in older adults becomes larger for cohorts vaccinated in the years following the switch of the childhood vaccine (Fig. 5B). In a 65-year-old cohort that is vaccinated 8 years after the switch to PCV20 in childhood vaccination, the ICER of PCV20 compared to no vaccination was €45,081/QALY gained (instead of €22,250/QALY gained if vaccinated in the year of the switch). From 3 years after the switch onwards, the ICER of 3xPPV23 compared to no vaccination was lower than that of PCV20, eventually becoming €34,571/QALY gained at 8 years post-switching. The ICER of PCV21 compared to no vaccination remained stable over time.

## Discussion

We assessed the cost-effectiveness of new available PCV15 and PCV20 and the in-development PCV21 for older adults in the Netherlands from a societal perspective. We accounted for indirect protection and serotype replacement from a switch to higher-valency PCVs in childhood vaccination. We found that the impact of the indirect effects from childhood vaccination depended on the serotype overlap between the vaccines used for children and older adults. With PCV10, PCV13, and PCV15 in children, PCV20 was the cost-effective strategy for a 65-year-old cohort at a conventional Dutch threshold of €20,000/QALY gained. This strategy dominated PPV23 and PCV15 and costed approximately €10,000/QALY gained compared to no vaccination. However, with PCV20 in children, the ICER of PCV20 compared to no vaccination for the 65-year-old cohort increased to €22,250/QALY gained, which would no longer be cost-effective. As indirect effects progressed over time, the cost-effectiveness of PCV20 for older adults further diminished, and PPV23 dominated PCV20 for cohorts vaccinated 3 years after the switch to PCV20 in children. In-development PCV21 offered the highest QALY gains for older adults, and its cost-effectiveness was minimally impacted by indirect effects from PCV20 in children, thanks to its coverage of 11 different serotypes compared to PCV20.

Several countries, including the USA, decided to recommend PCV20 for older adults. While currently considered a cost-effective approach, the decision becomes less straightforward if the use of PCV20 in childhood vaccination is anticipated. Then, the long-term cost-effectiveness of pneumococcal vaccination for older adults relies on a price reduction of PCV20, or the adoption of a vaccine covering different serotypes compared to PCV20, like PCV21. Although PPV23 could eventually become cost-saving compared to PCV20 in older adults after a switch to PCV20 in children, its cost-effectiveness (and that of PCV15 + PPV23) compared to no vaccination would also be substantially diminished. Alternatively, PPV23 for older adults could be maintained until PCV21 becomes available. However, there is uncertainty around the timing and magnitudes of indirect effects, while introducing PCV20 for older adults would offer immediate health benefits. Clearly, these decisions also depend on the availability and pricing of PCV21, which are currently unknown, as well as on the flexibility in switching vaccines.

Projecting the timing and magnitudes of indirect effects proves challenging due to its unpredictable nature. We based our assumptions on observations from childhood PCV10 and PCV13 programs [40, 41], where indirect protection on older adults began before serotype replacement. Indirect effects typically completed after 8 years, with no net effect on IPD incidence in non-vaccinated groups, though results varied between countries. Similar timescales were observed after PCV7 introduction [54]. Our results were sensitive to the magnitude of indirect effects; PCV20 became cost-effective to the €20,000/QALY gained threshold again if indirect protection from PCV20 in childhood vaccination decreased from 80 to 40%. If only half of the IPD and NIPP cases prevented by indirect protection from PCV20 in children were replaced with non-PCV20 serotypes, the cost-effectiveness of PCV21 compared to no vaccination diminished but remained below the €20,000/QALY gained threshold if equally priced to PCV20. Note that we did not vary the distribution or invasive capacity of replacement serotypes.

Previous studies also concluded that adopting PCV15 and PCV20 in childhood vaccination reduced the cost-effectiveness of PCV15, PCV20, or PCV21 for older adults through indirect protection. However, their conclusions on the cost-effectiveness remained unchanged, often explained by the omission of a “no vaccination” strategy. The cost-effectiveness between two vaccines could hardly change if both the intervention strategy (e.g., PCV20) and reference strategy (e.g., PPV23) become less cost-effective compared to no vaccination. Two studies found that when considering serotype replacement from PCV20 in childhood vaccination, the cost-effectiveness of PCV20 compared to PPV23 was minimally affected [20, 22]. This can be explained by the substantial overlap in serotypes between these vaccines. We demonstrated for PCV21 that serotype replacement could affect the cost-effectiveness of vaccinating older adults substantially if the vaccine offers broader protection against replacement serotypes.

Our study uses a static model that does not incorporate pneumococcal transmission dynamics. Dynamic modeling of pneumococcal carriage and transmission is complex, and competitive interactions between serotypes are not fully understood. Although dynamic transmission models are generally preferred for estimating indirect effects from childhood vaccination, the use of correction factors in a static model to adjust the incidence of infection in other age groups has often been applied successfully in economic evaluations of pneumococcal vaccines [55]. A recent dynamic modeling exercise for Germany estimated that the continued use of PCV13 in childhood vaccination could result in a 20% increase in overall IPD incidence among ≥ 60-year-olds between 2022 and 2031 [56]. This increase is explained by a continued rise in non-PCV13 serotypes. However, for some serotypes categories, the short-term predictions did not align well with observed IPD trends in Germany, illustrating the challenge of modeling serotype interactions. In our study, we assumed the IPD incidence in older adults to remain at the level observed in the period 2017–2019. If replacement leads to higher disease incidence in older adults than currently observed, our cost-effectiveness estimates could be considered conservative.

Our analysis has limitations. Firstly, uncertainties exist regarding the incidence and serotype distribution of hospitalized NIPP cases, the VE against NIPP, and vaccine protection duration. We addressed these with sensitivity analyses, yielding consistent outcomes across most scenarios. Secondly, we extrapolated the vaccine-type specific VE of PCV13 to PCV15, PCV20, and PCV21, despite lower immune responses in PCV15 and PCV20 for most serotypes shared with PCV13 [57, 58]. However, both PCV15 and PCV20 met the chosen non-inferiority criteria, and the clinical relevance of these lower immune responses is unclear. Thirdly, we focused on mortality within 30 days post-diagnosis, neglecting non-hospitalized deaths. Also, literature suggests that the risk of dying from IPD may persist for at least 1 year post-diagnosis [28], although with uncertain comorbidity factors. If included, the cost-effectiveness of pneumococcal vaccination would improve. Fourthly, we did not include the prevented burden of pneumococcal disease in primary care. However, a previous cost-effectiveness study of PCV13 for Dutch older adults in 2015 estimated that including vaccine effectiveness against NIPP in primary care changed the ICER of vaccinating adults aged 65–74 years from €8890/QALY gained to €8647/QALY gained [46]. Therefore, we do not anticipate that this omission will significantly impact our outcomes.

Strong points of our analysis include the utilization of high-quality data from one country for many model parameters. The VE estimation for PPV23, primarily derived from UK data, aligns well with early impact estimates of the PPV23 vaccination program for older adults in the Netherlands [59]. Also, we included a no vaccination strategy without requiring counterfactual calculations, since the Netherlands had no pneumococcal vaccination program for older adults until 2020. By taking indirect protection and serotype replacement from childhood vaccination into account, our study enhances the knowledge about the impact and cost-effectiveness of new higher-valency PCVs for older adults. This insight is valuable for all countries facing similar decisions in addressing pneumococcal diseases in older adults.

## Conclusions

Vaccinating older adults with PCV20 is the cost-effective strategy in the Netherlands if childhood vaccination occurs with PCV10, PCV13, or PCV15. However, once PCV20 is included in childhood vaccination, none of the currently available vaccines prove cost-effective to the €20,000/QALY gained threshold. To ensure long-term cost-effectiveness, the pneumococcal vaccine for older adults should either include serotypes not covered by the childhood vaccination program, like PCV21 aims to do, or become more affordable than its current pricing for individual use. We recommend that policy decisions aimed at reducing the burden of pneumococcal disease in the population at large incorporate both childhood and adult vaccination programs in a comprehensive economic assessment.

### Supplementary Information

**Additional file 1:** **Supplementary introduction: Table S1.** Summary of previous studies on the cost-effectiveness PCV15, PCV20 and PCV21 in adults. **Supplementary Methods: Figure S1.** Schematic overview of the model. **Figure S2.** The annual incidence of IPD cases in the Netherlands among adults aged ≥60 years per 100,000 inhabitants over the period 2004-2019 by serotype category. **Figure S3.** Fitted vaccine effectiveness of PPV23 against vaccine-type IPD and vaccine-type hospitalized NIPP at time of vaccination, across different vaccination ages. **Figure S4.** Fitted vaccine efficacy of PCVs against vaccine-type IPD and vaccine-type hospitalized NIPP at time of vaccination, across different vaccination ages. **Figure S5.** The number of QALYs lost by age of death. **Table S2.** Epidemiological parameter values. **Table S3.** Serotype categories distinguished and serotype distribution from IPD cases aged 60+ years in the Netherlands in 2019. **Table S4. **Parameter values of costs. **Table S5.** Parameter values of quality-adjusted life years (QALYs). **Table S6.** Parameters varied in the one-way sensitivity analyses and their input values. Supplementary Results: **Figure S6.** One-way sensitivity analysis of the cost-effectiveness of different pneumococcal vaccination strategies in a 65-years-old cohort while continuing PCV10 in children, or with a switch to PCV13, PCV15 or PCV20. **Figure S7.** Cost-effectiveness of various vaccination pneumococcal vaccination strategies compared to no vaccination by vaccination age, while PCV10 is continued in children. **Figure S8.** Cost-effectiveness of different pneumococcal vaccination strategies in older adults for different vaccination ages in the age-range 60 to 85 years, while varying the childhood vaccine between PCV10, PCV13, PCV15, and PCV20. **Table S7.** Clinical impact, number needed to vaccinate and cost-effectiveness of different pneumococcal vaccination strategies compared to no vaccination in a 65-year-old cohort, while continuing PCV10 in children. **Table S8.** Clinical impact, number needed to vaccinate and cost-effectiveness of different pneumococcal vaccination strategies compared to no vaccination in a 65-year-old cohort, while switching to PCV13 in children. **Table S9.** Clinical impact, number needed to vaccinate and cost-effectiveness of different pneumococcal vaccination strategies compared to no vaccination in a 65-year-old cohort, while switching to PCV15 in children. **Table S10.** Clinical impact, number needed to vaccinate and cost-effectiveness of different pneumococcal vaccination strategies compared to no vaccination in a 65-year-old cohort, while switching to PCV20 in children. **Table S11.** QALY losses, costs and incremental cost-effectiveness ratio of different vaccination strategies in a 65-year-olds cohort, while continuing PCV10 in children. **Table S12.** Incremental cost-effectiveness ratio of different vaccination strategies in a 65-year-olds cohort, while switching to PCV13 in children. **Table S13.** Incremental cost-effectiveness ratio of different vaccination strategies in a 65-year-olds cohort, while switching to PCV15 in children. **Table S14.** Incremental cost-effectiveness ratio of different vaccination strategies in a 65-year-olds cohort, while switching to PCV20 in children. **Table S15.** Clinical impact, number needed to vaccinate and cost-effectiveness of different pneumococcal vaccination strategies in a 60-year-old cohort, while PCV10 is continued in children. **Table S16.** Clinical impact, number needed to vaccinate and cost-effectiveness of different pneumococcal vaccination strategies in a 70-year-old cohort, while PCV10 is continued in children. **Table S17.** Clinical impact, number needed to vaccinate and cost-effectiveness of different pneumococcal vaccination strategies in a 75-year-old cohort, while PCV10 is continued in children. **Table S18.** Clinical impact, number needed to vaccinate and cost-effectiveness of different pneumococcal vaccination strategies in a 80-year-old cohort, while PCV10 is continued in children. **Table S19.** Clinical impact, number needed to vaccinate and cost-effectiveness of different pneumococcal vaccination strategies in a 85-year-old cohort, while PCV10 is continued in children. **Table S20.** Incremental cost-effectiveness ratio of different vaccination strategies in a 60-year-olds cohort, while remaining PC10 in children. **Table S21.** Incremental cost-effectiveness ratio of different vaccination strategies in a 70-year-olds cohort, while remaining PC10 in children. **Table S22.** Incremental cost-effectiveness ratio of different vaccination strategies in a 75-year-olds cohort, while remaining PC10 in children. **Table S23.** Incremental cost-effectiveness ratio of different vaccination strategies in a 80-year-olds cohort, while remaining PC10 in children. **Table S24.** Incremental cost-effectiveness ratio of different vaccination strategies in a 85-year-olds cohort, while remaining PC10 in children.

## Data Availability

All data generated or analyzed during this study are included in this published article and in Additional file 1. The model code is available from the corresponding author, PTdB, upon reasonable request.

## Abbreviations

IPD: Invasive pneumococcal disease
CAP: Community acquired pneumonia
PPV23: 23-Valent pneumococcal polysaccharide vaccine
PCV13: 13-Valent pneumococcal conjugate vaccine
PCV7: 7-Valent pneumococcal conjugate vaccine
PCV10: 10-Valent pneumococcal conjugate vaccine
PCV20: 20-Valent pneumococcal conjugate vaccine
PCV21: 21-Valent pneumococcal conjugate vaccine
QALY: Quality-adjusted life year
NIPP: Non-invasive pneumococcal pneumonia
VE: Vaccine effectiveness
