# Supplementary material for: Higher-valency pneumococcal conjugate vaccines in older adults, taking into account indirect effects from childhood vaccination: a cost-effectiveness study for the Netherlands
Source: BMC Med. 2024 Feb 16;22:69. doi: 10.1186/s12916-024-03277-3 (PMC10870576; doi:10.1186/s12916-024-03277-3)
Supplement: Supplementary file 1 — Additional file 1: Supplementary introduction: Table S1. Summary of previous studies on the cost-effectiveness PCV15, PCV20 and PCV21 in adults. Supplementary Methods: Figure S1. Schematic overview of the model. Figure S2. The annual incidence of IPD cases in the Netherlands among adults aged ≥60 years per 100,000 inhabitants over the period 2004-2019 by serotype category. Figure S3. Fitted vaccine effectiveness of PPV23 against vaccine-type IPD and vaccine-type hospitalized NIPP at time of vaccination, across different vaccination ages. Figure S4. Fitted vaccine efficacy of PCVs against vaccine-type IPD and vaccine-type hospitalized NIPP at time of vaccination, across different vaccination ages. Figure S5. The number of QALYs lost by age of death. Table S2. Epidemiological parameter values. Table S3. Serotype categories distinguished and serotype distribution from IPD cases aged 60+ years in the Netherlands in 2019. Table S4. Parameter values of costs. Table S5. Parameter values of quality-adjusted life years (QALYs). Table S6. Parameters varied in the one-way sensitivity analyses and their input values. Supplementary Results: Figure S6. One-way sensitivity analysis of the cost-effectiveness of different pneumococcal vaccination strategies in a 65-years-old cohort while continuing PCV10 in children, or with a switch to PCV13, PCV15 or PCV20. Figure S7. Cost-effectiveness of various vaccination pneumococcal vaccination strategies compared to no vaccination by vaccination age, while PCV10 is continued in children. Figure S8. Cost-effectiveness of different pneumococcal vaccination strategies in older adults for different vaccination ages in the age-range 60 to 85 years, while varying the childhood vaccine between PCV10, PCV13, PCV15, and PCV20. Table S7. Clinical impact, number needed to vaccinate and cost-effectiveness of different pneumococcal vaccination strategies compared to no vaccination in a 65-year-old cohort, while continuing PCV10 in children. Table S8. Cl [file 12916_2024_3277_MOESM1_ESM.docx]

# Additional file 1

Belonging to the manuscript:

**Higher-valency pneumococcal conjugate vaccines in older adults, taking into account indirect effects from childhood vaccination: A cost-effectiveness study for the Netherlands**

Pieter T. de Boer^1,*^, Cornelis H. van Werkhoven^1,2^, Albert Jan van Hoek^1^, Mirjam J. Knol^1^, Elisabeth A.M. Sanders^1,3^, Jacco Wallinga^1,4^, Hester E. de Melker^1^, Anneke Steens^1^

1. Center for Infectious Disease Control, National Institute for Public Health and the Environment (RIVM), the Netherlands
2. Julius Center for Health Sciences and Primary Care, University Medical Center Utrecht, Utrecht University, the Netherlands
3. Department of Pediatric Immunology and Infectious diseases, University Medical Center Utrecht, the Netherlands
4. Department of Biomedical Datasciences, Leiden University Medical Center, the Netherlands

# Supplementary introduction

Table S1: Summary of previous studies on the cost-effectiveness of 15-, 20- and 21-valent pneumococcal conjugate vaccines (PCV15, PCV20 and PCV21) in older adults, and whether indirect effects from childhood vaccination have been taken into account. Studies published before October 30, 2023, are included.

| Study; funding | Country; study population | Strategies; perspective | Consideration of indirect effects from PCV15/PCV20 in childhood vaccination;  magnitude of indirect effect | Cost-effectiveness outcomes;  **study conclusions** |
| --- | --- | --- | --- | --- |
| Smith 2021 [1];  independent | United States;  65-yo | PPV23+optional PCV13, PPV23, PCV15, PCV15+PPV23, PCV20, PCV20+PPV23; Health care payer | Indirect protection for PCV15- and PCV20-unique serotypes (*versus* PCV13) in sensitivity analysis; 50% incidence reduction; no serotype replacement | PCV20 dominates PCV15 strategies and costs US$172,491/QALY *versus* PPV23+optional PCV13. Indirect protection from PCV20 in childhood vaccination increased the ICER of PCV20 *versus* PPV23+optional PCV13 to US$449,260/QALY; **PPV23+optional PCV13 is the cost-effective strategy, especially after considering indirect protection from PCV20 in children** |
| Stoecker 2021 [2];  independent | United States;  50- or 65-yo cohorts | PPV23+optional PCV13, PCV15, PCV20, PCV15+PPV23, PCV20+PPV23;  Societal | Indirect protection for PCV15 unique serotypes (*versus* PCV13) from year 2 and PCV20-unique serotypes (*versus* PCV15) from year 3 in a sensitivity analysis; 4.1% incidence reduction per year; no serotype replacement | PCV20 dominates PPV23+optional PCV13, PCV15 and PCV15+PPV23. PCV20+PPV23 costs US$1,313,935/QALY *versus* PCV20 (65yo cohort). With indirect protection, PCV20 remains dominant *versus* PPV23+optional PCV13 (65yo cohort), but not on the long run; **PCV20 is the cost-effective strategy, also with short term indirect protection** |
| Smith 2022 [3];  independent | United States;  65-yo black and non-black cohort | PCV20, PCV15+PPV23, no vaccination; Health care payer | Not included | PCV20 costs US$169,540/QALY *versus* no vaccination and PCV15+PPV23 costs US$535,797/QALY *versus* PCV20 (black population); **PCV20 is the cost-effective strategy** |
| Mendes 2022 [4]; industry | England; ≥65-yo and high-risk 18- to 64-yo | PPV23, PCV20, PPV20+PPV23, PCV15+PPV23, PPV23+PPV23;  Health care payer | Indirect protection for PCV15-unique serotypes (*versus* PCV13) from year 2 and PCV20-unique serotypes (*versus* PCV15) from year 3 in the main analysis; 71-91% incidence reduction between year 1 and year 10, depending on age; no serotype replacement | PCV20 dominated PPV23, PPV23+PPV23, PCV15+PPV23. PCV20+PPV23 costs £686,948/QALY *versus* PCV20; **PCV20 is the cost-effective strategy, also with indirect protection** |
| Hoshi 2022 [5]; independent | Japan; ≥65-yo and high-risk 18- to 64-yo | PPV23, PCV15, PCV20; Health care payer | Not included | PCV20 dominated PPV23 and PCV15; **PCV20 is the cost-effective strategy** |
| Wateska 2022 [6]; Independent | United States;  50-yo black and non-black cohort | PPV23+optional PCV13, PCV20, PCV15+PPV23, revaccination at age 65y; Health care payer | Indirect protection for PCV15- and PCV20-unique serotypes (*versus* PCV13) in the sensitivity analysis; 0-100% incidence reduction; no serotype replacement | PCV15+PPV23 and PCV20 dominated PCV13+PPV23. PCV15+PPV23 costs US$104,723/QALY *versus* PCV20 (black cohort). Revaccination at 65-yo costs US$240,952/QALY *versus* no revaccination (black cohort). With indirect effects, PCV15+PPV23 and PCV20 also dominate PCV13+PPV23; **PCV15+PPV23 and PCV20 both cost-effective, also with indirect protection. Revaccination not cost-effective** |
| Olsen 2022 [7]; industry | Denmark; ≥65-yo and 18- to 64-yo with risk factor | PPV23, PCV20, PCV20+PPV23; Societal | Not included | PCV20+PPV23 dominated PPV23, PCV20 dominated PPV23;  **PCV20 is the cost-effective strategy, either as single dose or combined with PPV23** |
| Polistena 2022 [8]; industry | Italy; 65- to 74-yo | PCV13, PCV15, PCV20; Health care payer | Not included | PCV20 dominates PCV13, PCV20 costs €91/QALY *versus* PCV15; **PCV20 is the cost-effective strategy** |
| NACI 2023 ;  independent | Canada; 50-, 65- and 75-yo cohorts for northern Canada and rest of country | PPV23, PCV15, PCV20, PCV15+PPV23, PCV20+PPV23; Health care payer (main analysis) | Indirect protection for PCV15- and PCV20-unique serotypes (*versus* PCV13) from year 4 in sensitivity analysis; 50% incidence reduction; no serotype replacement | PCV20 dominated PCV15 and PCV15+PPV23 and costs Can$17,379/QALY compared to PPV23 (65-yo, rest of country). PCV20+PPV23 costs Can$80,344/QALY compared to PCV20. With indirect protection, PCV20 costs Can$21,316/QALY compared to PPV23 and PCV20+PPV23 costs Can$81,612/QALY (65-yo, rest of country); **PCV20 is the cost-effective strategy, also with indirect protection** |
| Wateska 2023 [9];  independent | United States; Black and non-black 65-yo cohort | PCV15+PPV23, PCV20, PCV21, no vaccination;  Health care payer | Indirect protection for PCV15-unique serotypes (*versus* PCV13) in the sensitivity analysis; 50% incidence reduction; no serotype replacement | PCV21 dominated PCV20 and PCV15+PPV23, and costs US$88,478/QALY compared to no vaccination (black 65-yo cohort). Indirect protection from PCV15 in childhood vaccination ren diminished the cost-effectiveness to US$97,952/QALY; **PCV21 is the cost-effective strategy, also with indirect protection from PCV15 in children** |
| Cantarero 2023 [10]; industry | Spain; ≥60-yo | PCV15+PPV23, PCV20; Health care payer | Not included | PCV20 dominated PCV15+PPV23; **PCV20 is the cost-effective strategy** |
| Restivo 2023 [11]; industry | Italy; low-risk ≥65-yo, moderate-risk 50- to 100-yo | PCV15+PPV23, PCV13+PPV23, PCV20, PPV20+PPV23, no vaccination;  Societal | Not included | Low-risk ≥65-year-olds: PCV20 dominates PCV15+PPV23 and PCV13+PPV23 and costs about €60,000/QALY *versus* no vaccination. PCV20+PPV23 costs about €175,000/QALY *versus* PCV20. Moderate-risk 50- to 100-year-olds: PCV20 dominated PCV15+PPV23, PCV13+ PPV23 and no vaccination. PCV20+PPV23 costs about €15,000/QALY *versus* PCV20; **Pneumococcal vaccination not cost-effective in low-risk older adults, PCV20+PPV23 the cost-effective strategy in high-risk older adults** |
| Mikkelsen 2023 [12];  industry | Norway; low-risk ≥65-yo and high-risk 18- to 99-yo | PPV23, PCV20;  Health care payer | Not included | PCV20 dominated PPV23; **PCV20 is the cost-effective strategy** |
| Kühne 2023 [13]; industry | Germany; ≥60-yo and moderate- / high-risk 18- to 59-yo | PPV23 / PCV13+PPV23, PCV15+PPV23, PCV20;  Health care payer (main analysis) | Indirect protection for PCV15-unique serotypes (*versus* PCV13) from year 2 and PCV20-unique serotypes (*versus* PCV15) from year 3 in the main analysis. Serotype replacement in the sensitivity analysis; 85-91% incidence reduction between year 1 and year 10, depending on age. Full serotype replacement | PCV20 dominated PPV23 / PCV13+PPV23 and PCV15+PPV23. PCV20 also dominated PPV23 / PCV13+PPV23 if no herd effects are applied or if full serotype replacement is considered; **PCV20 is the cost-effective strategy, also after including indirect protection and serotype replacement** |
| Gourzoulidis 2023 [14]; industry | Greece; ≥65-yo and high-risk 18-to 64-yo | PCV20, PCV15, PCV15+PPV23; Health care payers | Not included | PCV20 dominated PCV15 and PCV15+PPV23. **PCV20 is the cost-effective strategy** |
| Marbaix 2023 [15]; industry | Belgium, 65-84-yo, moderate-risk 50- to 64-yo, and high-risk 18- to 49-yo | No vaccination, PCV20, PCV15+PPV23; Health care payer (main analysis) | Indirect protection for PCV13-unique serotypes up to 2024, and for PCV15/PCV20 unique serotypes beyond 2024 in the main analysis. Serotype replacement in the sensitivity analysis; 50% incidence reduction over 5 years. Full serotype replacement | PCV20 costs €4,164/QALY *versus* no vaccination, and dominates PCV15+PPV23. If full serotype replacement is considered, PCV20 costs €4,353 *versus* no vaccination. **PCV20 is the cost-effective strategy, also after inclusion of serotype replacement.** |

ICER: Incremental cost-effectiveness ratio, PPV23: 23-valent pneumococcal polysaccharide vaccine, QALY: Quality-adjusted life year, yo: year-olds

# Supplementary Methods

## Model design

We adapted and updated a previously developed static multi-cohort model [16], consisting of single year age cohorts of individuals aged 60 years and older that were followed in annual time-steps. A schematic overview is presented in Figure S1. Each year, people had a probability of acquiring IPD or hospitalized NIPP based on the incidence corresponding to their age-group. We assumed all IPD patients to be hospitalized. Subsequently, IPD and hospitalized NIPP patients could die according to age- and outcome-specific case-fatality rates. Population sizes and all-cause mortality of 2019 from Statistics Netherlands were used to obtain realistic aging of the cohorts [17, 18]. Vaccination was assumed to be introduced with immediate effectiveness in the year 2025. To be able to determine the impact of vaccination, the hospitalized cases were distributed among different sets of pneumococcal serotypes covered by the various vaccines. The impact of pneumococcal vaccination was modelled as a risk reduction of the incidence of vaccine-type hospitalizations, which is the multiplication of vaccine uptake and vaccine-type vaccine effectiveness (VE). VE is modelled as a function of the VE at the moment of vaccination (take) and an annual waning rate. VE at take differs by vaccine type (PPV or PCV), outcome (IPD or NIPP), and age of vaccination. We did not include an effect of vaccination against lower-respiratory infections in primary care, given the uncertainty regarding whether pneumococcal vaccination provides significant protection against this outcome [19]. Furthermore, the relative contribution of pneumococcal disease burden in primary care on the cost-effectiveness outcomes has been shown to be limited [20].

Figure S1: Schematic overview of the model. Node M represents a Markov node that uses annual time steps to follow the cohort over increasing age. IPD: Invasive pneumococcal disease, NIPP: Non-invasive pneumococcal pneumonia.

## Epidemiology

Due to the response measures against the COVID-19 pandemic affecting the epidemiology of pneumococcal disease between 2020 and 2022 [21], we used epidemiological data from the Netherlands prior to 2020 to parameterize the model. Since the pneumococcal vaccination program for adults aged 60-79 years with PPV23 began in the Netherlands in 2020, these data pertain to an elderly population that is largely unvaccinated against pneumococcal disease.

An overview of the mean epidemiological input values and their corresponding distributions used for the probabilistic sensitivity analysis can be found in Table S2.

Table S2: Epidemiological parameter values that were used in in the main analysis (mean value) and their distributions used in the probabilistic sensitivity analysis.

| Parameter | Mean | Standard error | Distribution | Reference |
| --- | --- | --- | --- | --- |
| Projected cohort sizes in 2025 |  |  |  |  |
| 60y | 254,920 |  | Fixed | Estimated using [17, 18] |
| 65y | 228,209 |  | Fixed |  |
| 70y | 196,004 |  | Fixed |  |
| 75y | 175,878 |  | Fixed |  |
| 80y | 126,470 |  | Fixed |  |
| 85y | 77,765 |  | Fixed |  |
| IPD incidence (per 100,000) |  |  |  |  |
| 60-64y | 28.4 | 1.87 | Beta | Dutch sentinel surveillance, National Institute for Public Health and the Environment |
| 65-69y | 33.5 | 2.11 | Beta |  |
| 70-74y | 46.6 | 2.68 | Beta |  |
| 75-79y | 52.7 | 3.45 | Beta |  |
| 80-84y | 65.6 | 4.61 | Beta |  |
| 85-89y | 76.6 | 6.44 | Beta |  |
| ≥90y | 81.5 | 9.28 | Beta |  |
| Proportion invasive pneumonia given IPD |  |  |  |  |
| All ages | 79.8% | 2.38% | Beta | [22] |
| CAP incidence (per 100,000) |  |  |  |  |
| 60-64y | 225 | 2.66 | Beta | National hospital surveillance, Netherlands |
| 65-69y | 327 | 3.41 | Beta |  |
| 70-74y | 497 | 4.96 | Beta |  |
| 75-79y | 724 | 6.82 | Beta |  |
| 80-84y | 1,050 | 9.59 | Beta |  |
| 85-89y | 1,385 | 14.42 | Beta |  |
| ≥90y | 1,575 | 21.88 | Beta |  |
| Proportion of CAP caused by *S. pneumoniae* |  |  |  |  |
| All ages | 22.1% | 1.48% | Beta | [23] |
| Adjustment factor NIPP from 2012-2014 to 2017-2019 using IPD time trend |  |  |  |  |
| All ages | 0.97 |  | Fixed | Calculated |
| Case-fatality rate IPD |  |  |  |  |
| 60-69y | 9.9% | 0.93% | Beta | Calculated from [24] |
| 70-79y | 16.4% | 1.18% | Beta |  |
| 80-89y | 23.3% | 1.62% | Beta |  |
| ≥90y | 31.4% | 3.73% | Beta |  |
| Relative risk case-fatality hospitalized NIPP/IPD |  |  |  |  |
| All ages | 0.84 | 0.053 | Beta | [25] |

IPD: Invasive pneumococcal disease, NIPP: Non-invasive pneumococcal pneumonia, CAP: Community acquired pneumonia

Serotype-specific numbers of hospitalized IPD cases per 5-years age-group (60-64, 65-69, … ≥90) for the calendar years 2004-2019 were obtained from the Dutch sentinel IPD surveillance of the National Institute for Public Health and the Environment. Numbers were converted to incidences using national population sizes of the corresponding year [17], taking into account that the sentinel laboratories cover approximately 25% of the Dutch population. To avoid the impact of seasonal fluctuations, we used the average incidence of all serotypes by age-group in the years 2017-2019, which was partitioned into incidences per set of vaccine serotypes using the serotype distribution of all IPD cases aged ≥60 years of the most recent year of 2019 (Table S3 and Figure S2). IPD cases with missing serotype (3.4% of total) were divided among the different sets of (vaccine) serotypes proportionally to the distribution of the serotyped IPD cases.

Table S3: Serotype sets distinguished in the model and the serotype distribution from IPD cases aged ≥60 years in the Netherlands in 2019. Isolates with missing serotype were divided among the categories proportionally to the distribution of the serotyped isolates. ST: Serotype, PCV: pneumococcal conjugate vaccine, PPV: Pneumococcal polysaccharide vaccine

| ST category | Included ST | % of all IPD | PCV10 | PCV13 | PCV15 | PCV20 | PCV21 | PPV23 |
| --- | --- | --- | --- | --- | --- | --- | --- | --- |
| PCV10 minus 7F | 4, 6B, 9V, 14, 18C, 19F, 23F, 1, 5 | 2.8% | X | X | X | X |  | X |
| ST7F | 7F | 0.5% | X | X | X | X | X | X |
| ST3 | 3 | 8.6% |  | X | X | X | X | X |
| ST6A | 6A | 0.2% |  | X | X | X | X |  |
| ST6C (cross-protection from 6A)* | 6C | 4.4% |  | X | X | X | X |  |
| ST19A | 19A | 17.0% |  | X | X | X | X | X |
| PCV15 minus PCV13 | 22F, 33F | 11.0% |  |  | X | X | X | X |
| PCV20 minus PCV15 minus 15B | 8, 10A, 11A, 12F | 30.1% |  |  |  | X | X | X |
| ST15B* | 15B | 2.1% |  |  |  | X |  | X |
| PCV21/PPV23 minus PCV20 minus ST2 | 9N, 17F, 20 | 8.2% |  |  |  |  | X | X |
| ST2 | 2 | 0.0% |  |  |  |  |  | X |
| PCV21 minus PCV20/PPV23 | 15A, 15C, 16F, 23A, 23B, 24F, 31, 35B | 11.0% |  |  |  |  | X |  |
| Non-vaccine types minus ST6C | Other STs + non-typeable pneumococci | 4.2% |  |  |  |  |  |  |
| Total coverage |  | 100% | 3.3% | 33.6% | 44.5% | 76.7% | 90.9% | 80.2% |

PCV: Pneumococcal conjugate vaccine, PPV: pneumococcal polysaccharide vaccine, ST: Serotype
* For PCV13, it has been established that the serotype 6A antigen provides clinical effectiveness in protecting against 6C disease [26].


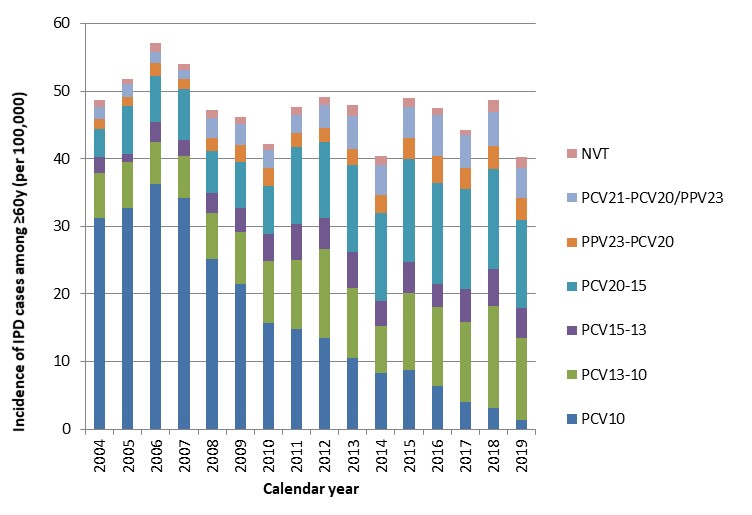


Figure S2: The annual incidence of IPD cases in the Netherlands among adults aged ≥60 years per 100,000 inhabitants over the period 2004-2019 by serotype category. For visualization reasons we merged the serotype categories of Table S3 as follows: PCV10: PCV10 minus 7F+ST7F, PCV13-10: ST6A+ST3+ST19A+ST6C (cross-protection from 6A), PCV20-15: PCV20 minus PCV15 minus 15B+ST15B, PPV23-PCV20: PCV21/PPV23 minus PCV20 minus ST2+ST2. PCV: Pneumococcal conjugate vaccine, PPV: pneumococcal polysaccharide vaccine, IPD: Invasive pneumococcal disease.

The annual incidence of NIPP hospitalizations was based on multiple data sources. Annual numbers of hospitalizations from all-cause community-acquired pneumonia (CAP) (International Classification of Diseases, ICD-10-AM codes J9-J18) per 5-years age-group (60-64, 65-69, …, ≥90) were obtained from national Dutch hospital surveillance data for the years 2012-2014, and converted to incidence using national population sizes. From these all-cause CAP hospitalizations, we assumed 22.1% to be caused by *S. pneumoniae*, as observed in the placebo-arm of the CAPITA trial [23]. Subsequently, the incidence of pneumococcal CAP was adjusted for the time-trend of pneumococcal disease from 2012-2014 to 2017-2019, based on the change in incidence of IPD hospitalizations among adults aged ≥60 years across this period. Eventually, the incidence of NIPP hospitalizations was obtained by subtracting the incidence of invasive pneumococcal pneumonia hospitalizations from the incidence of pneumococcal CAP cases, taking into account that 79.8% of the IPD cases are invasive pneumonia cases in the Dutch surveillance data [22]. To validate the estimated incidence of NIPP, we compared the ratio between the incidence of NIPP and IPD in the age-range 65-79 years with the ratio of NIPP and IPD cases observed in the placebo-arm of the CAPITA trial [23], which appeared to be reasonably similar (1.6 in our analysis *versus* 1.7 in CAPITA). In absence of data on the serotype distribution of NIPP hospitalizations, we also used the serotype distribution of IPD cases for NIPP.

Thirty-day case-fatality rates for IPD by age-group (60-69, 70-79, 80-89, ≥90) were obtained by re-analyzing data of Vestjens et al. [24] from January 2008 to May 2011 and from January 2013 to May 2016. The case-fatality rates of NIPP were obtained by multiplying the age-specific case-fatality rates of IPD with 0.84, based on the ratio of 30-day case fatality rates between IPD cases and non-IPD CAP hospitalized patients from a Dutch study conducted in the period 2008-2013 [25].

## Indirect effects from higher-valency PCVs in children on older adults

In the Netherlands, a PCV7 program for children started in 2006, switching to a PCV10 program in 2011. The vaccination coverage of pneumococcal vaccination in Dutch children has continuously been ≥90% [27]. Figure S2 shows the IPD incidence by serotype category for the ≥60-year-old population in the period 2004-2019. The incidence of PCV10 serotypes had almost disappeared in 2019, while the contribution of the other serotypes has grown, resulting in an small net reduction in IPD incidence since pre-PCV10. Furthermore, the contributions of the different serotype groups tended to stabilize, as the incidence of PCV10 serotypes had nearly disappeared. Data from countries using PCV10 or PCV13 showed that indirect effects of pneumococcal childhood vaccination programs on the epidemiology in older adults more or less stabilized after eight years [26, 28, 29], which would be around 2019 in the Netherlands. Therefore, we assumed that the serotype distribution of pneumococcal disease in adults aged in the ≥60-year-old population has reached a steady state if PCV10 was continued in the childhood vaccination.

In case of a switch to PCV13, PCV15 or PCV20 in the childhood vaccination program, we assumed indirect effects on older adults. The assumptions on the magnitudes of these indirect effects and the time-frame in which these effects occur were based on trends observed in the IPD incidence in countries using PCV10 and/or PCV13 in their childhood vaccination program, found by the multi-country studies PSERENADE and SpIDnet [14-16]. In these studies, indirect protection started earlier than serotype replacement and the serotype distributions tended to stabilize after eight years. PSERENADE data shows that the IPD incidence from PCV10-included serotypes among ≥65-year-olds decreased by over 75% at eight years after the implementation of the childhood PCV program. This was followed by an increase in IPD incidence from non-vaccine serotypes due to serotype replacement. The net impact on overall IPD incidence varied between countries, ranging from no change to a 50% decline. In the Netherlands, no significant change in IPD incidence among ≥65-year-olds was observed in the period 2008-2019, despite an over 90% reduction in IPD incidence from PCV10 serotypes. Therefore, we assumed the following:

1. Due to indirect protection, the incidence of childhood vaccine serotypes added to PCV10 decreases by 80% in older adults. This decrease occurs linearly, starting one year post-implementation, and completing 8 years post-implementation. No indirect protection was assumed for serotype 3. Cross-protection to serotype 6C was included.
2. Due to serotype replacement, the incidence of non-childhood vaccine serotypes increases in older adults until the incidence of all serotypes reached the pre-indirect effects level (i.e. 100% replacement). This increase occurs linearly, starting three years post-implementation and completing 8 years post-implementation. The relative contribution of different non-vaccine serotypes remained constant during the increase.

The new steady state reached eight years post-switch is maintained for the remaining time-horizon. The magnitudes of indirect effects were applied uniformly for both IPD cases and NIPP hospitalizations. Given the uncertainty surrounding the assumed magnitudes of indirect effects, we conducted sensitivity analyses on these parameters. As modelling indicates that an increase in vaccine valency could reduce indirect effects [30], we also explored a scenario with 40% indirect protection. For serotype replacement we examined a scenario in which 50% of disappeared serotypes were replaced.

## Vaccination

The vaccine uptake in older adults was assumed at 70%, approximately matching the actual estimated uptake of PPV23 vaccination in the Netherlands in the calendar years 2020 and 2021 [31, 32].

For PPV23, the VE against vaccine-type IPD at time of vaccination was estimated by age of vaccination using two observational cohort studies from the UK [33, 34]. These studies provided VE estimates for age-groups 65-74 years, 75-84 years and ≥85 years within two years after vaccination (Figure S3). We used an average age of 69 years for the age-group 65-74 years, 79 years for the age-group 75-84 years and 88 years for the age-group ≥85 years. Subsequently, we fitted a third-order polynomial function through the data points of the two studies, using the size of the confidence intervals as weights. To determine the VE of PPV23 against vaccine-type hospitalized NIPP, we relied on an estimate from a recent meta-analysis of observational studies with data up to 5 years after vaccination for the age-group 65-74 years (26% [95% CI: -0.05- 0.49 ] [35]). Given the absence of age-specific data for this outcome, we assigned this estimate to the age of 69 years and used the age-related trend of the VE observed for IPD. The VE at age 65 years is also used for the age range 60-64 years. In case of a negative VE in the fit, a VE of 0 was used in the analysis. A revaccination with PPV23 after 5 years was assumed to have the same VE at time of vaccination as a first dose would have had at that age; hence, no hypo-responsiveness was assumed, as suggested to occur with revaccination within five years [36].

For PCVs, the VE against vaccine-type IPD and vaccine-type NIPP at time of vaccination were derived from the CAPITA study [23], a large placebo-controlled randomized clinical trial of PCV13 in persons aged ≥65 years conducted in the Netherlands in the period 2008-2014. A *post hoc* analysis of CAPITA data showed a significant decline in VE against vaccine-type IPD or NIPP with increasing age at vaccination [37]. Therefore, we used single-year age-specific VE estimates against vaccine-type IPD and NIPP from Cox proportional hazards models that were applied on the modified intention to treat data of persons aged 65-89 years of CAPITA (Figure S4). For the age-range 60-64 years we assumed the VE to be the same as the VE for persons aged 65 years. In case of a negative VE in the fit, a VE of 0 was used in the analysis. For the probabilistic sensitivity analysis we obtained 1,000 runs from the Cox proportional hazards model used to estimate the 95% confidence intervals. Runs showing an increasing VE with increasing age were removed, as these were thought to be unrealistic. To account for the relatively healthy population in the CAPITA trial, the vaccine-type specific VE estimates were multiplied by a factor 0.9. The VE against vaccine-type IPD as estimated for PCV13 was extrapolated to the additional vaccines types covered by PCV15 and PCV20. We assumed that PCVs also provide cross-protection against the non-vaccine serotype 6C, acknowledging evidence on cross-protection via serotype 6A [26].

The duration of protection has been assumed to be in line with a previous cost-effectiveness analysis for the Netherlands [16]. The VE for PPV23 remained constant for two years and then linearly decreased to 0% at five years post-vaccination. For PCV, no evidence of waning immunity was reported throughout the 4 years of follow-up in the CAPITA study, but vaccine efficacy data after this period is lacking. Therefore, we assumed no waning over the first four years after vaccination, followed by a linear waning to 0% after 15 years post-vaccination.

For combination strategies of a PCV with PPV23 in older adults, we modelled the VE over time separately for each vaccine. For serotypes present in both vaccines we used the highest VE at each time-step; hence, we did not assume additional efficacy against serotypes present in both vaccines. In practice, the VE of PCV was higher than PPV in most of the analysis years, except for a short period after a revaccination with PPV23 at year 6 and year 11.

For both vaccines we assumed that during the simulations of the probabilistic sensitivity analysis simulation, the VE could not be negative; those simulations assumed 0% effectiveness.


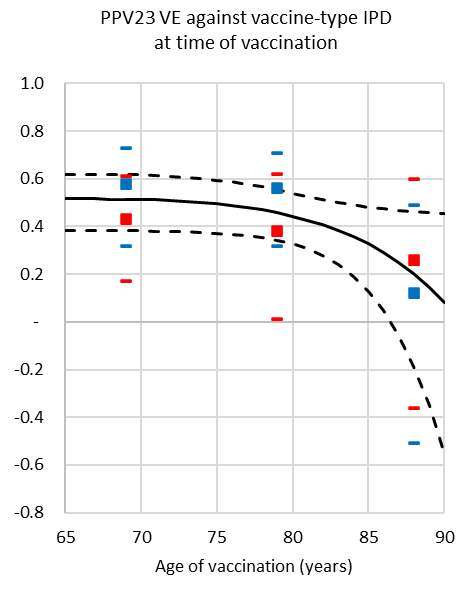

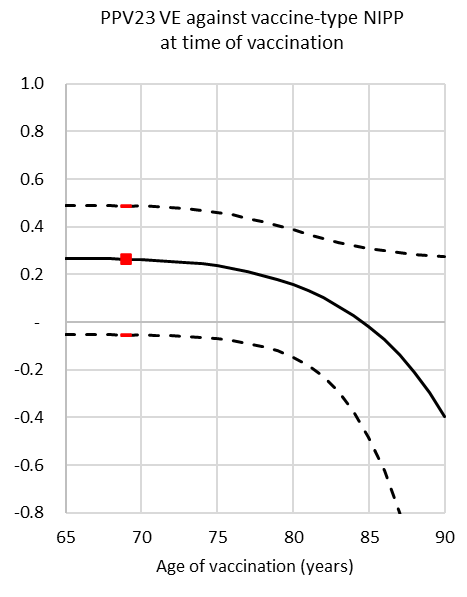


Figure S3: Fitted vaccine effectiveness (VE) of PPV23 against vaccine-type IPD and vaccine-type hospitalized NIPP at time of vaccination, across different vaccination ages. The straight line represents the median estimate, and the dashed lines the lower and upper limits of the 95%-confidence intervals. The VE against IPD is fitted to data from Andrews et al. (red markers) and Djennad et al. (blue markers). The VE against NIPP is based on a VE estimate for the age-group 65-74 years from a meta-analysis by Farrar et al. (red markers), and extrapolated across age using the trend of the VE of PPV23 against IPD. In case the fit would results in a negative VE, the cost-effectiveness analysis uses a VE of 0%. PPV: pneumococcal polysaccharide vaccine, IPD: Invasive pneumococcal disease, NIPP: non-invasive pneumococcal pneumonia.


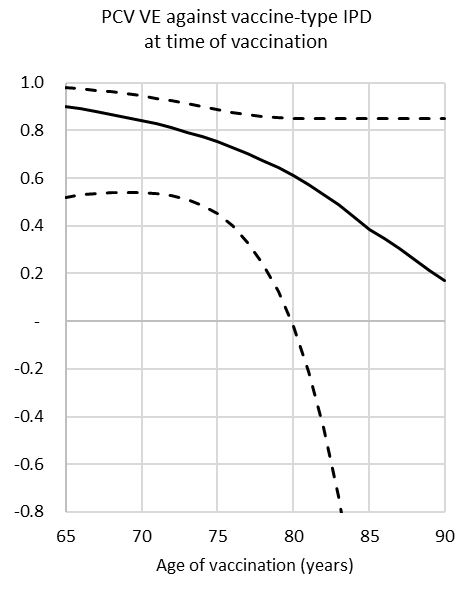

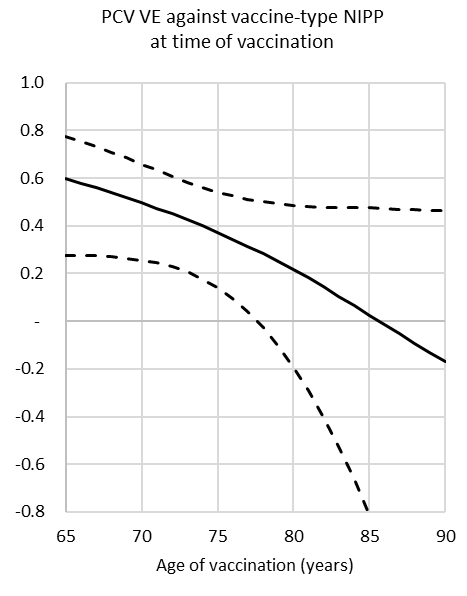


Figure S4: Fitted vaccine efficacy (VE) of PCVs against vaccine-type IPD and vaccine-type hospitalized NIPP at time of vaccination, across different vaccination ages. The fit uses CAPITA trial data of participants aged below 90 years (see [37], data shared by C.H. van Werkhoven). The straight line represents the median estimate, and the dashed lines the lower and upper limits of the 95%-confidence intervals. For the cost-effectiveness analysis, an adjusted factor of 0.9 is applied to account for the relative healthier population of the CAPITA trial. In case the fit would results in a negative VE, the analysis uses a VE of 0%. PCV: Pneumococcal conjugate vaccine, IPD: Invasive pneumococcal disease, NIPP: non-invasive pneumococcal pneumonia.

## Costs

Mean input values and their distributions used for the probabilistic sensitivity analysis are shown in Table S4. The cost year of the analysis was 2021, and all costs were inflated to this price year using the Dutch consumer price index (all sectors) [38]. Vaccine prices of PPV23, PCV15, PCV20 were obtained from a Dutch price list for individual use [39]. The vaccine price of PCV21 is unknown, and we assumed it to be the same as PCV20. The administration cost per vaccination was based on the fee a general practitioner received in 2021 for the invitation and administration of PPV23 in the national vaccination program [40]. Age-specific hospitalization costs due to IPD and NIPP were based on a Dutch study conducted in parallel with the CAPITA trial [25]. The hospitalization costs for NIPP were based on costs from patients with a non-invasive CAP episode. Indirect health care cost, which encompass non-pneumococcal related medical costs in gained life years of averted deaths, were considered only in a sensitivity analysis. These costs were estimated using the Practical Application to Include Disease Costs (PAID) tool, version 3.0 [41]. Patient costs due to IPD and hospitalized NIPP were based on data from a cluster-randomized cross-over trial of antibiotic treatment strategies [42]. We used the weighted average of the three study groups. Estimates of productivity losses resulting from IPD, hospitalized NIPP and death following hospitalization were obtained from a prior cost-effectiveness analysis of PCV13 for older adults in the Netherlands [20]. This study estimated the productivity losses of deaths with the friction method [43], assuming a maximum amount of 160 work days lost. We did not consider productivity losses above the age of 75 years.

Table S4: Parameter values of costs that were used in the main analysis (mean value) and their distributions used in the probabilistic sensitivity analysis.

| Parameter | Mean | Standard error | Distribution | Reference |
| --- | --- | --- | --- | --- |
| *Vaccination costs* |  |  |  |  |
| Vaccine cost per dose |  |  |  |  |
| PPV23 | €25.94 |  | Fixed | [39] |
| PCV13 | €74.72 |  | Fixed | [39] |
| PCV15 | €74.73 |  | Fixed | [39] |
| PCV20 | €82.95 |  | Fixed | [39] |
| PCV21 | Equal to PCV20 |  | Fixed | Assumption |
| Administration costs | €21.00 |  | Fixed | [44] |
| *Direct healthcare costs* |  |  |  |  |
| IPD in-patient |  |  |  |  |
| 60-64y | Equal to 65-74y |  |  | Assumption |
| 65-74y | €13,373 | €666 | Lognormal | [25] |
| 75-84y | €18,961 | €1,318 | Lognormal |  |
| ≥85y | €8,595 | €1,336 | Lognormal |  |
| CAP in-patient |  |  |  |  |
| 60-64y | Equal to 65-74y |  |  | Assumption |
| 65-74y | €9,807 | €25 | Lognormal | [25] |
| 75-84y | €9,690 | €24 | Lognormal |  |
| ≥85y | €7,093 | €20 | Lognormal |  |
| *Indirect healthcare costs (in sensitivity analysis only)* |  |  |  |  |
| Healthcare costs in gained life years | Age-specific |  | Fixed | Estimated using [41] |
| *Direct non-healthcare costs (patient costs)* |  |  |  |  |
| IPD/NIPP hospitalization | €144 | €12 | Gamma | [42] |
| *Indirect non-healthcare costs (productivity losses)* |  |  |  |  |
| IPD hospitalization |  |  |  |  |
| 60-64y | €4,145 | € 300 | Gamma | [20] |
| 65-74y | €330 | € 24 | Gamma |  |
| 75+y | €0 |  | Fixed |  |
| NIPP hospitalization |  |  |  |  |
| 60-64y | €2,747 | € 66 | Gamma | [20] |
| 65-74y | €330 | € 8 | Gamma |  |
| ≥75y | €0 |  | Fixed |  |
| Death (either IPD or NIPP, additional to hospitalization costs) |  |  |  |  |
| 60-64y | €17,937 |  | Fixed | [20] |
| 65-74y | €3,116 |  | Fixed |  |
| ≥75y | €0 |  | Fixed |  |

PCV: Pneumococcal conjugate vaccine, PPV: pneumococcal polysaccharide vaccine, IPD: Invasive pneumococcal disease, NIPP: non-invasive pneumococcal pneumonia

[20, 25, 40, 42]

## Health effects

Quality-adjusted life year (QALY) loss inputs and their distribution for the probabilistic sensitivity analysis are shown in Table S5. The QALY losses of both IPD and hospitalized NIPP were based on a Dutch study that assessed the utility loss among hospitalized non-fatal CAP cases during the acute phase and the first month of the illness using the EuroQol five dimensional instrument (EQ-5D) [9]. QALYs lost due to premature death was estimated using the life-expectancy of a general Dutch person at that age [17], adjusted for age-specific Dutch general population utilities measured with EQ-5D [26] (Figure S5).

Table S5: Parameter values of quality-adjusted life years (QALYs) lost that were used in the main analysis (mean value) and their distributions used in the probabilistic sensitivity analysis.

| Parameter | Mean | Standard error | Distribution | Reference |
| --- | --- | --- | --- | --- |
| QALY loss IPD / NIPP hospitalization | 0.0709 | 0.0200 | Beta | [9] |
| Life years lost due to IPD/NIPP mortality | Age-specific, see Figure S6 |  | Fixed | [17] |
| General population utilities |  |  |  |  |
| 60-69y | 0.839 | 0.0142 | Beta | [26] |
| ≥70y | 0.852 | 0.0144 | Beta |  |

IPD: Invasive pneumococcal disease, NIPP: non-invasive pneumococcal pneumonia, QALY: Quality-adjusted life year


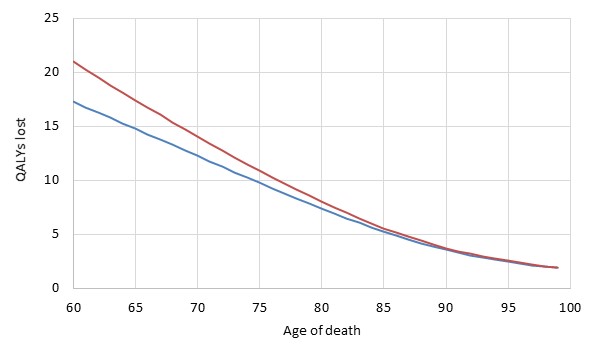


Figure S5: The number of QALYs lost by age of death. The red line shows the undiscounted QALY loss and the blue line the discounted QALY loss at 1.5% per year. QALY: Quality-adjusted life year

## Sensitivity analysis

We performed several one-way sensitivity analyses, in which one parameter was varied while the other parameters were kept fixed. The assumptions are shown in Table S6.

Table S6: Parameters varied in the one-way sensitivity analyses and their input values

| Parameter | Base case | Sensitivity analysis |
| --- | --- | --- |
| % CAP hospitalizations caused by *S. pneumoniae* | 22.1% | 30% |
| VE of PCV | Age-specific, mean value | Age-specific, lower bound of 95% confidence interval |
|  |  | Age-specific, upper bound of 95% confidence interval |
| VE of PPV23 | Age-specific, mean value | Age-specific, lower bound of 95% confidence interval |
|  |  | Age-specific, upper bound of 95% confidence interval |
| VE against ST3 | Equal VE for all serotypes | No VE against serotype 3 |
| VE against hospitalized NIPP | Included | No VE against hospitalized NIPP |
| Waning immunity for PCV | VE 4 years stable, linear decline to 0% at 15 years after vaccination | VE 4 years stable, linear decline to 0% at 10 years after vaccination* |
|  |  | VE 8 years stable, linear decline to 0% at 15 years after vaccination |
| Waning immunity for PPV23 | VE 2 years stable, linear decline to 0% at 5 years after vaccination | VE 5 years stable, and instant to 0% at 5 years after vaccination |
| QALY loss hospitalized IPD/NIPP | 0.706, based on the disease period and one month follow-up | 0.15, based on the disease period and one year follow-up [45] |
| Indirect health care costs | No inclusion of indirect health care costs | Inclusion of indirect health care costs as presented in Table S3 |
| Perspective | Societal perspective | Health care payer’s perspective (only including direct medical costs) |
| Discounting | 4%/1.5% per year for costs/QALYs | No discounting |
|  |  | 4%/4% per year for costs/QALYs |
| Administration costs €10 | €21 | €10 |
| PPV23 price per dose | €25.94 | 13% discount (€22.50) |
|  |  | 50% discount (possible price reduction following a tender [46]) |
| PCV price per dose | Varies per vaccine (List price – see Table S3) | 13% discount, varies per vaccine |
|  |  | 50% discount (possible price reduction following a tender [46]), varies per vaccine |

* For this analysis we reduced the time-horizon from 15 years to 10 years to avoid that a third dose of PPV23 after 10 years is included in the comparison.
IPD: Invasive pneumococcal disease, NIPP: non-invasive pneumococcal pneumonia, PCV: Pneumococcal conjugate vaccine, PPV: pneumococcal polysaccharide vaccine, QALY: Quality-adjusted life year.

[45, 46]

## References

1. Smith KJ, Wateska AR, Nowalk MP, Lin CJ, Harrison LH, Schaffner W, Zimmerman RK. Higher-Valency Pneumococcal Conjugate Vaccines: An Exploratory Cost-Effectiveness Analysis in U.S. Seniors. Am J Prev Med 2021, 61(1):28-36.

2. Stoecker C. Economic assessment of PCV15 & PCV20 2021 <https://stacks.cdc.gov/view/cdc/109109>. Accessed at 12 June 2023.

3. Smith KJ, Wateska AR, Nowalk MP, Lin CJ, Harrison LH, Schaffner W, Zimmerman RK. Cost-Effectiveness of Newly Recommended Pneumococcal Vaccination Strategies in Older Underserved Minority Adults in the USA. Infect Dis Ther 2022, 11(4):1683-1693.

4. Mendes D, Averin A, Atwood M, Sato R, Vyse A, Campling J, Weycker D, Slack M, Ellsbury G, Mugwagwa T. Cost-effectiveness of using a 20-valent pneumococcal conjugate vaccine to directly protect adults in England at elevated risk of pneumococcal disease. Expert Rev Pharmacoecon Outcomes Res 2022, 22(8):1285-1295.

5. Hoshi SL, Shono A, Seposo X, Okubo R, Kondo M. Cost-effectiveness analyses of 15- and 20-valent pneumococcal conjugate vaccines for Japanese elderly. Vaccine 2022, 40(49):7057-7064.

6. Wateska AR, Patricia Nowalk M, Lin CJ, Harrison LH, Schaffner W, Zimmerman RK, Smith KJ. Cost-effectiveness of revised US pneumococcal vaccination recommendations in underserved minority adults < 65-years-old. Vaccine 2022, 40(50):7312-7320.

7. Olsen J, Schnack H, Skovdal M, Vietri J, Mikkelsen MB, Poulsen PB. Cost-effectiveness of 20-valent pneumococcal conjugate vaccine in Denmark compared with PPV23. J Med Econ 2022, 25(1):1240-1254.

8. Polistena B, Icardi G, Orsi A, Spandonaro F, Di Virgilio R, d'Angela D. Cost-Effectiveness of Vaccination with the 20-Valent Pneumococcal Conjugate Vaccine in the Italian Adult Population. Vaccines (Basel) 2022, 10(12).

9. Wateska AR, Nowalk MP, Lin CJ, Harrison LH, Schaffner W, Zimmerman RK, Smith KJ. Cost-effectiveness of an in-development adult-formulated pneumococcal vaccine in older US adults. Vaccine 2023, 41(30):4431-4437.

10. Cantarero D, Ocana D, Onieva-Garcia MA, Rodriguez-Garcia J, Galvez P, Mendez C, Crespo C, Lopez-Ibanez de Aldecoa A. Cost-utility analysis of the use of the 20-valent anti-pneumococcal vaccine (PCV20) in adults older than 60 years in Spain. Vaccine 2023, 41(36):5342-5349.

11. Restivo V, Baldo V, Sticchi L, Senese F, Prandi GM, Pronk L, Owusu-Edusei K, Johnson KD, Ignacio T. Cost-Effectiveness of Pneumococcal Vaccination in Adults in Italy: Comparing New Alternatives and Exploring the Role of GMT Ratios in Informing Vaccine Effectiveness. Vaccines (Basel) 2023, 11(7).

12. Malene BM, Oyvind H, Tor M, David NM, Jens O, Nanna VK, Jeffrey V. Cost-effectiveness of 20-valent pneumococcal conjugate vaccine compared with 23-valent pneumococcal polysaccharide vaccine among adults in a Norwegian setting. Cost Eff Resour Alloc 2023, 21(1):52.

13. Kuhne F, Achtert K, Puschner F, Urbanski-Rini D, Schiller J, Mahar E, Friedrich J, Atwood M, Sprenger R, Vietri J *et al*. Cost-effectiveness of use of 20-valent pneumococcal conjugate vaccine among adults in Germany. Expert Rev Vaccines 2023, 22(1):921-932.

14. Gourzoulidis G, Barmpouni M, Kossyvaki V, Vietri J, Tzanetakos C. Health and economic outcomes of 20-valent pneumococcal conjugate vaccine compared to 15-valent pneumococcal conjugate vaccine strategies for adults in Greece. Front Public Health 2023, 11:1229524.

15. Marbaix S, Mignon A, Taelman A, Averin A, Atwood M, Vietri J. Cost-utility of 20-valent pneumococcal conjugate vaccine compared to no vaccination and recommended alternative vaccines among Belgian adults. Expert Rev Vaccines 2023.

16. Thorrington D, van Rossum L, Knol M, de Melker H, Rumke H, Hak E, van Hoek AJ. Impact and cost-effectiveness of different vaccination strategies to reduce the burden of pneumococcal disease among elderly in the Netherlands. PLoS One 2018, 13(2):e0192640.

17. Statistics Netherlands. Population; sex, age and marital status, Jan. 1 2021 <https://opendata.cbs.nl/statline/#/CBS/nl/dataset/7461BEV/table?fromstatweb>. Accessed at 7 June 2023.

18. Statistics Netherlands. Life expectancy; sex, age (by year and five-year period) 2022 <https://opendata.cbs.nl/statline/#/CBS/nl/dataset/37360ned/table>. Accessed at 7 June 2023.

19. van Werkhoven CH, Bolkenbaas M, Huijts SM, Verheij TJM, Bonten MJM. Effects of 13-valent pneumococcal conjugate vaccination of adults on lower respiratory tract infections and antibiotic use in primary care: secondary analysis of a double-blind randomized placebo-controlled study. Clin Microbiol Infect 2021, 27(7):995-999.

20. Mangen MJ, Rozenbaum MH, Huijts SM, van Werkhoven CH, Postma DF, Atwood M, van Deursen AM, van der Ende A, Grobbee DE, Sanders EA *et al*. Cost-effectiveness of adult pneumococcal conjugate vaccination in the Netherlands. Eur Respir J 2015, 46(5):1407-1416.

21. Steens A, Knol MJ, Freudenburg-de Graaf W, de Melker HE, van der Ende A, van Sorge NM. Pathogen- and Type-Specific Changes in Invasive Bacterial Disease Epidemiology during the First Year of the COVID-19 Pandemic in The Netherlands. Microorganisms 2022, 10(5).

22. Wagenvoort GH, Sanders EA, de Melker HE, van der Ende A, Vlaminckx BJ, Knol MJ. Long-term mortality after IPD and bacteremic versus non-bacteremic pneumococcal pneumonia. Vaccine 2017, 35(14):1749-1757.

23. Bonten MJM, Huijts AM, Bolkenbaas M, Webber C, Patterson S, Gault S, van Werkhoven CH, van Deursen AMM, Sanders EAM, Verheij TJM *et al*. Polysaccharide Conjugate Vaccine against Pneumococcal Pneumonia in Adults. NEJM 2015, 372:1114-1125.

24. Vestjens SMT, Sanders EAM, Vlaminckx BJ, de Melker HE, van der Ende A, Knol MJ. Twelve years of pneumococcal conjugate vaccination in the Netherlands: Impact on incidence and clinical outcomes of invasive pneumococcal disease. Vaccine 2019, 37(43):6558-6565.

25. Vissink CE, Huijts SM, de Wit GA, Bonten MJ, Mangen MJ. Hospitalization costs for community-acquired pneumonia in Dutch elderly: an observational study. BMC Infect Dis 2016, 16:466.

26. Savulescu C, Krizova P, Valentiner-Branth P, Ladhani S, Rinta-Kokko H, Levy C, Mereckiene J, Knol M, Winje BA, Ciruela P *et al*. Effectiveness of 10 and 13-valent pneumococcal conjugate vaccines against invasive pneumococcal disease in European children: SpIDnet observational multicentre study. Vaccine 2022, 40(29):3963-3974.

27. National Institute for Public Health and the Environment. National Vaccination Program; type of vaccination, region [In Dutch] 2023 <https://statline.rivm.nl/#/RIVM/nl/dataset/50117NED/table>. Accessed at 19 January 2024.

28. Knoll MD, on behalf of the PSERENADE Team. Conclusions from the PSERENADE Project: Implications for Pneumococcal Vaccine Policy and What is Happening Next 2021 <https://www.meningitis.org/getmedia/0a2e175e-4e48-401e-8482-e2b2a9c5bf11/Maria-Knoll?disposition=attachment>. Accessed at 1 October 2023.

29. Hanquet G, Krizova P, Dalby T, Ladhani SN, Nuorti JP, Danis K, Mereckiene J, Knol MJ, Winje BA, Ciruela P *et al*. Serotype Replacement after Introduction of 10-Valent and 13-Valent Pneumococcal Conjugate Vaccines in 10 Countries, Europe. Emerg Infect Dis 2022, 28(1):137-138.

30. Masala GL, Lipsitch M, Bottomley C, Flasche S. Exploring the role of competition induced by non-vaccine serotypes for herd protection following pneumococcal vaccination. J R Soc Interface 2017, 14(136).

31. Heins M, Hooiveld M, Korevaar J. Vaccinatiegraad Nationaal Programma Pneumokokkenvaccinatie Volwassenen 2020: monitor in het kort 2021 <https://www.nivel.nl/sites/default/files/bestanden/1004085.pdf>. Accessed at 1 September 2023.

32. Heins M, Korevaar J, Knottnerus B, Hooiveld M. Monitor vaccinatiegraad nationaal programma pneumokokkenvaccinatie volwassenen (NPPV) 2021 2022 <https://www.nivel.nl/sites/default/files/bestanden/1004275.pdf>. Accessed at 1 September 2023.

33. Andrews NJ, Waight PA, George RC, Slack MP, Miller E. Impact and effectiveness of 23-valent pneumococcal polysaccharide vaccine against invasive pneumococcal disease in the elderly in England and Wales. Vaccine 2012, 30(48):6802-6808.

34. Djennad A, Ramsay ME, Pebody R, Fry NK, Sheppard C, Ladhani SN, Andrews NJ. Effectiveness of 23-Valent Polysaccharide Pneumococcal Vaccine and Changes in Invasive Pneumococcal Disease Incidence from 2000 to 2017 in Those Aged 65 and Over in England and Wales. EClinicalMedicine 2018, 6:42-50.

35. Farrar JL, Childs L, Ouattara M, Akhter F, Britton A, Pilishvili T, Kobayashi M. Systematic Review and Meta-Analysis of the Efficacy and Effectiveness of Pneumococcal Vaccines in Adults. Pathogens 2023, 12(5).

36. Croucher NJ, Lochen A, Bentley SD. Pneumococcal Vaccines: Host Interactions, Population Dynamics, and Design Principles. Annu Rev Microbiol 2018, 72:521-549.

37. van Werkhoven CH, Huijts SM, Bolkenbaas M, Grobbee DE, Bonten MJ. The Impact of Age on the Efficacy of 13-valent Pneumococcal Conjugate Vaccine in Elderly. Clin Infect Dis 2015, 61(12):1835-1838.

38. Statistics Netherlands. Consumer prices; price index 2015=100 2023 <https://opendata.cbs.nl/#/CBS/en/dataset/83131ENG/table>. Accessed at 11 April 2023.

39. National Health Care Institute. Medicijnkosten.nl 2022 <https://www.medicijnkosten.nl/>. Accessed at 1 October 2022.

40. Government Gazette of the Kingdom of the Netherlands. Regulation of the State Secretary for Health, Welfare and Sport of 3 March 2020, reference 1649720-201966-PG, amending the Public Health Subsidy Scheme in connection with vaccination against pneumococcal disease and several other adjustments 2020 <https://zoek.officielebekendmakingen.nl/stcrt-2020-14106.html>. Accessed at 11 April 2023.

41. van Baal PH, Wong A, Slobbe LC, Polder JJ, Brouwer WB, de Wit GA. Standardizing the inclusion of indirect medical costs in economic evaluations. Pharmacoeconomics 2011, 29(3):175-187.

42. van Werkhoven CH, Postma DF, Mangen MJ, Oosterheert JJ, Bonten MJ, group C-Ss. Cost-effectiveness of antibiotic treatment strategies for community-acquired pneumonia: results from a cluster randomized cross-over trial. BMC Infect Dis 2017, 17(1):52.

43. National Health Care Institute. Guideline for conducting health economic evaluations in health care 2016 <https://www.zorginstituutnederland.nl/over-ons/publicaties/publicatie/2016/02/29/richtlijn-voor-het-uitvoeren-van-economische-evaluaties-in-de-gezondheidszorg>. Accessed at 1 October 2022.

44. Stichting Nationaal Programma Grieppreventie. Vergoeding 2022 <https://www.snpg.nl/article/griep-ha-afronden/declareren-griepvaccinaties/vergoeding/#:~:text=Vergoedingen%20in%202022%3A,het%20NPG%20en%2Fof%20NPPV>. Accessed at 1 October 2022.

45. Mangen MJ, Huijts SM, Bonten MJ, de Wit GA. The impact of community-acquired pneumonia on the health-related quality-of-life in elderly. BMC Infect Dis 2017, 17(1):208.

46. Zeevat F, van der Schans J, Boersma WG, Boersma C, Postma MJ. Cost-effectiveness analysis on elderly pneumococcal vaccination in the Netherlands: Challenging the Dutch Health Council's advice. Vaccine 2019, 37(43):6282-6284.

# Supplementary Results

## Clinical impact and cost-effectiveness, varying childhood vaccine

### Clinical impact and cost-effectiveness tables compared to no pneumococcal vaccination, age 65 years

Table S7 presents the reduction in IPD cases and NIPP hospitalizations, the number needed to vaccinate per prevented outcome, and the incremental cost-effectiveness ratio (ICER) compared to no vaccination for different pneumococcal vaccination strategies in a 65-year-old cohort over a 15-year period, assuming an unchanged childhood vaccination program with PCV10. Table S8, Table S9, and Table S10 show the results the results when taking into account indirect effects resulting from a change in the childhood vaccination program from PCV10 to PCV13, PCV15, or PCV20, respectively.

Table S7: Clinical impact, number needed to vaccinate and cost-effectiveness of different pneumococcal vaccination strategies compared to no vaccination in a 65-year-old cohort, while continuing PCV10 in children. The analysis assumed an uptake of 70% in older adults.

| Outcome | No vaccination | Difference to no vaccination | | | | | |
| --- | --- | --- | --- | --- | --- | --- | --- |
|  |  | 3xPPV23 | PCV15 | PCV20 | PCV15+ 3xPPV23 | PCV20+ 3xPPV23 | PCV21 |
| PPV23 administered | - | 450,282 | - | - | 443,458 | 443,469 | - |
| PCV administered | - | - | 159,875 | 159,875 | 159,875 | 159,875 | 159,875 |
| *Clinical outcomes* |  |  |  |  |  |  |  |
| IPD hospitalizations | 1,360 | -274 | -208 | -358 | -365 | -430 | -424 |
| NIPP hospitalizations | 199 | -40 | -28 | -48 | -52 | -60 | -57 |
| IPD deaths | 2,281 | -227 | -214 | -369 | -335 | -413 | -437 |
| NIPP deaths | 290 | -29 | -25 | -44 | -41 | -49 | -52 |
| *Costs* |  |  |  |  |  |  |  |
| PPV23 vaccination | - | 17.75 | - | - | 16.83 | 16.83 | - |
| PCV vaccination | 15.53 | - | 15.30 | 16.62 | 15.30 | 16.62 | 16.62 |
| IPD medical costs | 19.73 | -3.19 | -2.39 | -4.12 | -4.19 | -4.95 | -4.89 |
| NIPP medical costs | 0.15 | -1.98 | -1.92 | -3.30 | -2.96 | -3.67 | -3.91 |
| IPD patient costs | 0.29 | -0.03 | -0.02 | -0.04 | -0.04 | -0.05 | -0.05 |
| NIPP patient costs | 0.54 | -0.03 | -0.03 | -0.05 | -0.04 | -0.05 | -0.06 |
| IPD productivity losses | 1.07 | -0.11 | -0.11 | -0.19 | -0.16 | -0.20 | -0.22 |
| NIPP productivity losses | - | -0.11 | -0.12 | -0.20 | -0.17 | -0.22 | -0.24 |
| *QALY loss* |  |  |  |  |  |  |  |
| IPD hospitalization | 87 | -18 | -14 | -23 | -24 | -28 | -28 |
| NIPP hospitalization | 119 | -12 | -12 | -21 | -18 | -23 | -25 |
| IPD death | 1,917 | -400 | -305 | -525 | -520 | -615 | -622 |
| NIPP death | 2,213 | -233 | -229 | -394 | -343 | -432 | -467 |
| *Total* |  |  |  |  |  |  |  |
| Costs | 37.30 | 12.29 | 10.71 | 8.71 | 24.57 | 24.30 | 7.25 |
| Vaccination | - | 17.75 | 15.30 | 16.62 | 32.13 | 33.45 | 16.62 |
| IPD | 16.21 | -3.34 | -2.53 | -4.35 | -4.39 | -5.20 | -5.16 |
| NIPP | 21.09 | -2.12 | -2.06 | -3.55 | -3.18 | -3.95 | -4.21 |
| QALY loss | 4,336 | -662 | -559 | -963 | -906 | -1,098 | -1,141 |
| IPD | 2,004 | -417 | -318 | -548 | -544 | -643 | -650 |
| NIPP | 2,332 | -245 | -241 | -415 | -362 | -455 | -492 |
| *Relative to no vaccination* |  |  |  |  |  |  |  |
| NNV per IPD case avoided |  | 651 | 859 | 499 | 488 | 415 | 421 |
| NNV per NIPP hospitalization avoided |  | 4,461 | 6,365 | 3,697 | 3,457 | 2,993 | 3,119 |
| NNV per death avoided |  | 699 | 746 | 433 | 474 | 386 | 365 |
| ICER (€/QALY gained) |  | 18,559 | 19,162 | 9,051 | 27,128 | 22,134 | 6,352 |

ICER: Incremental cost-effectiveness ratio, IPD: Invasive pneumococcal disease, NIPP: Non-invasive pneumococcal pneumonia, NNV: Number needed to vaccinate, PCV: Pneumococcal conjugate vaccine, PPV: pneumococcal polysaccharide vaccine, QALY: Quality-adjusted life year

Table S8: Clinical impact, number needed to vaccinate and cost-effectiveness of different pneumococcal vaccination strategies compared to no vaccination in a 65-year-old cohort, while switching to PCV13 in children. The analysis assumed an uptake of 70% in older adults.

| Outcome | No vaccination | Difference to no vaccination | | | | | |
| --- | --- | --- | --- | --- | --- | --- | --- |
|  |  | 3xPPV23 | PCV15 | PCV20 | PCV15+ 3xPPV23 | PCV20+ 3xPPV23 | PCV21 |
| PPV23 administered | - | 450,283 | - | - | 443,457 | 443,468 | - |
| PCV administered | - | - | 159,875 | 159,875 | 159,875 | 159,875 | 159,875 |
| *Clinical outcomes* |  |  |  |  |  |  |  |
| IPD hospitalizations | 1,346 | -271 | -164 | -335 | -340 | -410 | -412 |
| NIPP hospitalizations | 2,263 | -224 | -165 | -344 | -305 | -393 | -426 |
| IPD deaths | 197 | -40 | -21 | -45 | -48 | -57 | -56 |
| NIPP deaths | 288 | -28 | -19 | -41 | -37 | -47 | -50 |
| *Costs* |  |  |  |  |  |  |  |
| PPV23 vaccination | - | 17.75 | - | - | 16.83 | 16.83 | - |
| PCV vaccination | 15.36 | - | 15.30 | 16.62 | 15.30 | 16.62 | 16.62 |
| IPD medical costs | 19.55 | -3.16 | -1.91 | -3.87 | -3.90 | -4.74 | -4.76 |
| NIPP medical costs | 0.15 | -1.96 | -1.49 | -3.09 | -2.69 | -3.49 | -3.81 |
| IPD patient costs | 0.29 | -0.03 | -0.02 | -0.04 | -0.04 | -0.05 | -0.05 |
| NIPP patient costs | 0.53 | -0.03 | -0.02 | -0.05 | -0.04 | -0.05 | -0.06 |
| IPD productivity losses | 1.06 | -0.11 | -0.09 | -0.18 | -0.15 | -0.19 | -0.22 |
| NIPP productivity losses | - | -0.11 | -0.09 | -0.19 | -0.16 | -0.21 | -0.23 |
| *QALY loss* |  |  |  |  |  |  |  |
| IPD hospitalization | 86 | -17 | -11 | -22 | -22 | -27 | -27 |
| NIPP hospitalization | 118 | -12 | -10 | -20 | -17 | -22 | -24 |
| IPD death | 1,898 | -395 | -239 | -491 | -482 | -587 | -605 |
| NIPP death | 2,193 | -230 | -178 | -368 | -311 | -410 | -455 |
| *Total* |  |  |  |  |  |  |  |
| Costs | 36.94 | 12.36 | 11.69 | 9.21 | 25.16 | 24.72 | 7.50 |
| Vaccination | - | 17.75 | 15.30 | 16.62 | 32.13 | 33.45 | 16.62 |
| IPD | 16.04 | -3.30 | -2.02 | -4.08 | -4.09 | -4.97 | -5.02 |
| NIPP | 20.91 | -2.10 | -1.60 | -3.32 | -2.89 | -3.75 | -4.10 |
| QALY loss | 4,294 | -655 | -437 | -901 | -831 | -1,045 | -1,111 |
| IPD | 1,984 | -412 | -250 | -513 | -504 | -614 | -632 |
| NIPP | 2,310 | -242 | -187 | -388 | -327 | -432 | -479 |
| *Relative to no vaccination* |  |  |  |  |  |  |  |
| NNV per IPD case avoided |  | 591 | 977 | 478 | 470 | 390 | 388 |
| NNV per NIPP hospitalization avoided |  | 712 | 969 | 464 | 524 | 407 | 376 |
| NNV per death avoided |  | 2,352 | 3,961 | 1,869 | 1,881 | 1,539 | 1,507 |
| ICER (€/QALY gained) |  | 18,882 | 26,730 | 10,228 | 30,258 | 23,650 | 6,750 |

ICER: Incremental cost-effectiveness ratio, IPD: Invasive pneumococcal disease, NIPP: Non-invasive pneumococcal pneumonia, NNV: Number needed to vaccinate, PCV: Pneumococcal conjugate vaccine, PPV: pneumococcal polysaccharide vaccine, QALY: Quality-adjusted life year

Table S9: Clinical impact, number needed to vaccinate and cost-effectiveness of different pneumococcal vaccination strategies compared to no vaccination in a 65-year-old cohort, while switching to PCV15 in children. The analysis assumed an uptake of 70% in older adults.

| Outcome | No vaccination | Difference to no vaccination | | | | | |
| --- | --- | --- | --- | --- | --- | --- | --- |
|  |  | 3xPPV23 | PCV15 | PCV20 | PCV15+ 3xPPV23 | PCV20+ 3xPPV23 | PCV21 |
| PPV23 administered | - | 450,284 | - | - | 443,456 | 443,468 | - |
| PCV administered | - | - | 159,875 | 159,875 | 159,875 | 159,875 | 159,875 |
| *Clinical outcomes* |  |  |  |  |  |  |  |
| IPD hospitalizations | 1,339 | -262 | -130 | -319 | -320 | -395 | -405 |
| NIPP hospitalizations | 2,254 | -217 | -127 | -327 | -282 | -377 | -418 |
| IPD deaths | 197 | -38 | -16 | -43 | -45 | -55 | -55 |
| NIPP deaths | 287 | -27 | -14 | -38 | -34 | -45 | -49 |
| *Costs* |  |  |  |  |  |  |  |
| PPV23 vaccination | - | 17.75 | - | - | 16.83 | 16.83 | - |
| PCV vaccination | 15.28 | - | 15.30 | 16.62 | 15.30 | 16.62 | 16.62 |
| IPD medical costs | 19.47 | -3.05 | -1.54 | -3.69 | -3.68 | -4.57 | -4.67 |
| NIPP medical costs | 0.15 | -1.89 | -1.16 | -2.93 | -2.48 | -3.35 | -3.75 |
| IPD patient costs | 0.29 | -0.03 | -0.02 | -0.04 | -0.04 | -0.05 | -0.05 |
| NIPP patient costs | 0.52 | -0.03 | -0.02 | -0.04 | -0.04 | -0.05 | -0.06 |
| IPD productivity losses | 1.06 | -0.11 | -0.07 | -0.17 | -0.14 | -0.18 | -0.21 |
| NIPP productivity losses | - | -0.10 | -0.07 | -0.18 | -0.14 | -0.20 | -0.23 |
| *QALY loss* |  |  |  |  |  |  |  |
| IPD hospitalization | 85 | -17 | -9 | -21 | -21 | -26 | -27 |
| NIPP hospitalization | 117 | -12 | -8 | -19 | -15 | -21 | -24 |
| IPD death | 1,888 | -382 | -189 | -467 | -452 | -565 | -595 |
| NIPP death | 2,182 | -222 | -139 | -350 | -286 | -394 | -447 |
| *Total* |  |  |  |  |  |  |  |
| Costs | 36.76 | 12.54 | 12.42 | 9.57 | 25.62 | 25.05 | 7.65 |
| Vaccination | - | 17.75 | 15.30 | 16.62 | 32.13 | 33.45 | 16.62 |
| IPD | 15.95 | -3.19 | -1.63 | -3.90 | -3.85 | -4.80 | -4.93 |
| NIPP | 20.81 | -2.03 | -1.25 | -3.15 | -2.66 | -3.60 | -4.03 |
| QALY loss | 4,273 | -633 | -344 | -857 | -774 | -1,006 | -1,092 |
| IPD | 1,973 | -399 | -198 | -488 | -473 | -591 | -622 |
| NIPP | 2,299 | -234 | -147 | -369 | -301 | -415 | -471 |
| *Relative to no vaccination* |  |  |  |  |  |  |  |
| NNV per IPD case avoided |  | 611 | 1,226 | 502 | 500 | 404 | 395 |
| NNV per NIPP hospitalization avoided |  | 738 | 1,254 | 490 | 568 | 424 | 382 |
| NNV per death avoided |  | 2,440 | 5,268 | 1,976 | 2,025 | 1,603 | 1,533 |
| ICER (€/QALY gained) |  | 19,824 | 36,065 | 11,173 | 33,090 | 24,908 | 7,007 |

ICER: Incremental cost-effectiveness ratio, IPD: Invasive pneumococcal disease, NIPP: Non-invasive pneumococcal pneumonia, NNV: Number needed to vaccinate, PCV: Pneumococcal conjugate vaccine, PPV: pneumococcal polysaccharide vaccine, QALY: Quality-adjusted life year

Table S10: Clinical impact, number needed to vaccinate and cost-effectiveness of different pneumococcal vaccination strategies compared to no vaccination in a 65-year-old cohort, while switching to PCV20 in children. The analysis assumed an uptake of 70% in older adults.

| Outcome | No vaccination | Difference to no vaccination | | | | | |
| --- | --- | --- | --- | --- | --- | --- | --- |
|  |  | 3xPPV23 | PCV15 | PCV20 | PCV15+ 3xPPV23 | PCV20+ 3xPPV23 | PCV21 |
| PPV23 administered | - | 450,283 | - | - | 443,454 | 443,460 | - |
| PCV administered | - | - | 159,875 | 159,875 | 159,875 | 159,875 | 159,875 |
| *Clinical outcomes* |  |  |  |  |  |  |  |
| IPD hospitalizations | 1,319 | -195 | -130 | -204 | -250 | -290 | -383 |
| NIPP hospitalizations | 2,227 | -158 | -127 | -197 | -222 | -264 | -396 |
| IPD deaths | 194 | -28 | -16 | -25 | -34 | -39 | -52 |
| NIPP deaths | 284 | -19 | -14 | -21 | -26 | -30 | -47 |
| *Costs* |  |  |  |  |  |  |  |
| PPV23 vaccination | - | 17.75 | - | - | 16.83 | 16.83 | - |
| PCV vaccination | 15.04 | - | 15.30 | 16.62 | 15.30 | 16.62 | 16.62 |
| IPD medical costs | 19.21 | -2.30 | -1.54 | -2.43 | -2.91 | -3.39 | -4.42 |
| NIPP medical costs | 0.14 | -1.40 | -1.16 | -1.81 | -1.98 | -2.38 | -3.54 |
| IPD patient costs | 0.28 | -0.02 | -0.02 | -0.03 | -0.03 | -0.03 | -0.05 |
| NIPP patient costs | 0.51 | -0.02 | -0.02 | -0.03 | -0.03 | -0.04 | -0.05 |
| IPD productivity losses | 1.04 | -0.08 | -0.07 | -0.11 | -0.12 | -0.14 | -0.20 |
| NIPP productivity losses | - | -0.08 | -0.07 | -0.11 | -0.12 | -0.14 | -0.22 |
| *QALY loss* |  |  |  |  |  |  |  |
| IPD hospitalization | 84 | -13 | -9 | -14 | -16 | -19 | -25 |
| NIPP hospitalization | 115 | -9 | -8 | -12 | -12 | -15 | -22 |
| IPD death | 1,859 | -285 | -189 | -295 | -355 | -412 | -563 |
| NIPP death | 2,152 | -165 | -139 | -216 | -230 | -278 | -423 |
| *Total* |  |  |  |  |  |  |  |
| Costs | 36.23 | 13.85 | 12.42 | 12.10 | 26.96 | 27.33 | 8.14 |
| Vaccination | - | 17.75 | 15.30 | 16.62 | 32.13 | 33.45 | 16.62 |
| IPD | 15.69 | -2.41 | -1.63 | -2.57 | -3.05 | -3.56 | -4.67 |
| NIPP | 20.54 | -1.50 | -1.25 | -1.94 | -2.13 | -2.55 | -3.81 |
| QALY loss | 4,211 | -471 | -344 | -537 | -613 | -724 | -1,033 |
| IPD | 1,943 | -298 | -198 | -309 | -371 | -431 | -588 |
| NIPP | 2,267 | -174 | -147 | -228 | -242 | -293 | -445 |
| *Relative to no vaccination* |  |  |  |  |  |  |  |
| NNV per IPD case avoided |  | 819 | 1,226 | 783 | 639 | 552 | 417 |
| NNV per NIPP hospitalization avoided |  | 1,011 | 1,254 | 812 | 720 | 605 | 404 |
| NNV per death avoided |  | 3,397 | 5,268 | 3,458 | 2,660 | 2,327 | 1,622 |
| ICER (€/QALY gained) |  | 29,381 | 36,065 | 22,550 | 43,956 | 37,738 | 7,876 |

ICER: Incremental cost-effectiveness ratio, IPD: Invasive pneumococcal disease, NIPP: Non-invasive pneumococcal pneumonia, NNV: Number needed to vaccinate, PCV: Pneumococcal conjugate vaccine, PPV: pneumococcal polysaccharide vaccine, QALY: Quality-adjusted life year

### Incremental cost-effectiveness tables, age 65 years

Table S11 shows the incremental costs and QALY losses, and ICERs of the various pneumococcal vaccination strategies in a 65-year-old cohort over a 15-year time-horizon, assuming an unchanged childhood vaccination program with PCV10. Table S12, Table S13 and Table S14 show the results when a change in children from PCV10 to PCV13, PCV15 or PCV20, respectively, was assumed.

Table S11: QALY losses, costs and incremental cost-effectiveness ratio of different vaccination strategies in a 65-year-olds cohort, while continuing PCV10 in children.

| Schedule | Total QALY loss | Total costs (€, millions) | Comparator | Incremental QALYs | Incremental costs (€, millions) | ICER (€/QALY gained) |
| --- | --- | --- | --- | --- | --- | --- |
| No vaccination | 4,336 | 37.3 |  |  |  |  |
| PCV15 | 3,776 | 49.3 | No vaccination | 559 | 12.03 | Dominated |
| 3xPPV23 | 3,673 | 49.6 | No vaccination | 662 | 12.29 | Dominated |
| PCV15+3xPPV23 | 3,430 | 63.2 | No vaccination | 906 | 25.88 | Dominated |
| PCV20 | 3,373 | 46.0 | No vaccination | 963 | 8.71 | 9,051 |
| PCV20+3xPPV23 | 3,238 | 61.6 | PCV20 | 135 | 15.59 | 115,412 |
| *PCV21** | *3,194* | *44.6* | *No vaccination* | *1,141* | *7.25* | *6,352* |

* As PCV21 is currently under development, we assumed its vaccine price to be equal to PCV20. Therefore, this strategy was excluded from the incremental comparison and only indicatively compared with no vaccination. ICER: Incremental cost-effectiveness ratio, PPV: pneumococcal polysaccharide vaccine, PCV: Pneumococcal conjugate vaccine, QALY: Quality-adjusted life year.

Table S12: Incremental cost-effectiveness ratio of different vaccination strategies in a 65-year-olds cohort, while switching to PCV13 in children.

| Schedule | Total QALY loss | Total costs (€, millions) | Comparator | Incremental QALYs | Incremental costs (€, millions) | ICER (€/QALY gained) |
| --- | --- | --- | --- | --- | --- | --- |
| No vaccination | 4,294 | 36.94 |  |  |  |  |
| PCV15 | 3,857 | 48.63 | No vaccination | 437 | 11.69 | Dominated |
| 3xPPV23 | 3,639 | 49.30 | No vaccination | 655 | 12.36 | Dominated |
| PCV15+3xPPV23 | 3,462 | 62.10 | No vaccination | 831 | 25.16 | Dominated |
| PCV20 | 3,393 | 46.16 | No vaccination | 901 | 9.21 | 10,228 |
| PCV20+3xPPV23 | 3,248 | 61.67 | PCV20 | 145 | 24.72 | 107,314 |
| *PCV21** | *3,183* | *44.44* | *No vaccination* | *1,111* | 7.50 | *6,750* |

* As PCV21 is currently under development, we assumed its vaccine price to be equal to PCV20. Therefore, this strategy was excluded from the incremental comparison and only indicatively compared with no vaccination. ICER: Incremental cost-effectiveness ratio, PPV: pneumococcal polysaccharide vaccine, PCV: Pneumococcal conjugate vaccine, QALY: Quality-adjusted life year.

Table S13: Incremental cost-effectiveness ratio of different vaccination strategies in a 65-year-olds cohort, while switching to PCV15 in children.

| Schedule | Total QALY loss | Total costs (€, millions) | Comparator | Incremental QALYs | Incremental costs (€, millions) | ICER (€/QALY gained) |
| --- | --- | --- | --- | --- | --- | --- |
| No vaccination | 4,273 | 36.76 |  |  |  |  |
| PCV15 | 3,928 | 49.18 | No vaccination | 344 | 12.42 | Dominated |
| 3xPPV23 | 3,640 | 49.30 | No vaccination | 633 | 12.54 | Dominated |
| PCV15+3xPPV23 | 3,498 | 62.38 | No vaccination | 774 | 25.62 | Dominated |
| PCV20 | 3,416 | 46.33 | No vaccination | 857 | 9.57 | 11,173 |
| PCV20+3xPPV23 | 3,267 | 61.81 | PCV20 | 149 | 15.48 | 103,800 |
| *PCV21** | *3,180* | *44.42* | *No vaccination* | *1,092* | *7.65* | *7,007* |

* As PCV21 is currently under development, we assumed its vaccine price to be equal to PCV20. Therefore, this strategy was excluded from the incremental comparison and only indicatively compared with no vaccination. ICER: Incremental cost-effectiveness ratio, PPV: pneumococcal polysaccharide vaccine, PCV: Pneumococcal conjugate vaccine, QALY: Quality-adjusted life year.

Table S14: Incremental cost-effectiveness ratio of different vaccination strategies in a 65-year-olds cohort, while switching to PCV20 in children.

| Schedule | Total QALY loss | Total costs (€, millions) | Comparator | Incremental QALYs | Incremental costs (€, millions) | ICER (€/QALY gained) |
| --- | --- | --- | --- | --- | --- | --- |
| No vaccination | 4,211 | 36.23 | No vaccination |  |  |  |
| PCV15 | 3,866 | 48.65 | No vaccination | 344 | 12.42 | Dominated |
| 3xPPV23 | 3,739 | 50.08 | No vaccination | 471 | 13.85 | Dominated |
| PCV20 | 3,674 | 48.33 | No vaccination | 537 | 12.10 | 22,550 |
| PCV15+3xPPV23 | 3,597 | 63.19 | PCV20 | 613 | 26.96 | Dominated |
| PCV20+3xPPV23 | 3,486 | 63.56 | PCV20 | 188 | 15.23 | 81,193 |
| *PCV21** | *3,177* | *44.37* | *No vaccination* | *1,033* | *8.14* | *7,876* |

* As PCV21 is currently under development, we assumed its vaccine price to be equal to PCV20. Therefore, this strategy was excluded from the incremental comparison and only indicatively compared with no vaccination. ICER: Incremental cost-effectiveness ratio, PPV: pneumococcal polysaccharide vaccine, PCV: Pneumococcal conjugate vaccine, QALY: Quality-adjusted life year.

### One-way sensitivity analysis, age 65 years

The one-way sensitivity analysis showed that the cost-effectiveness results sensitive to the vaccine price, the VE and its waning rate, and the proportion of CAP caused by *S. pneumoniae* (Figure S6). However, for the childhood vaccination scenarios with PCV10, PCV13 or PCV15, the dominating strategy only changed to PPV23 in case the VE was changed. For the scenario with PCV20 being used in children, the dominating strategy also changed to PPV23 in case of slower decrease of the VE of PPV23 or a lower price for PPV23 was assumed.


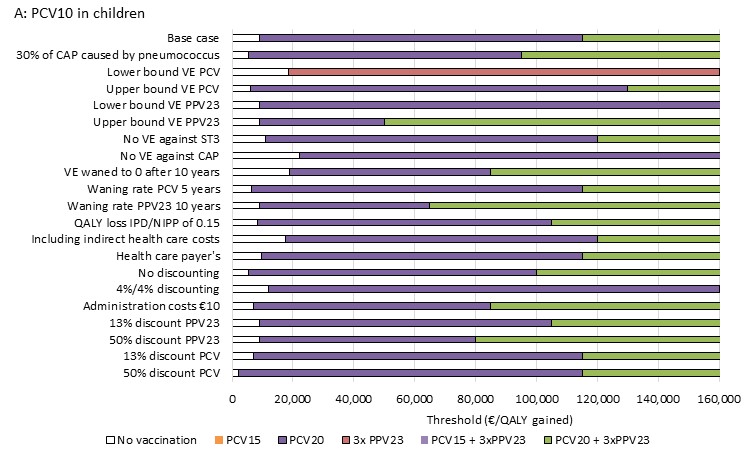


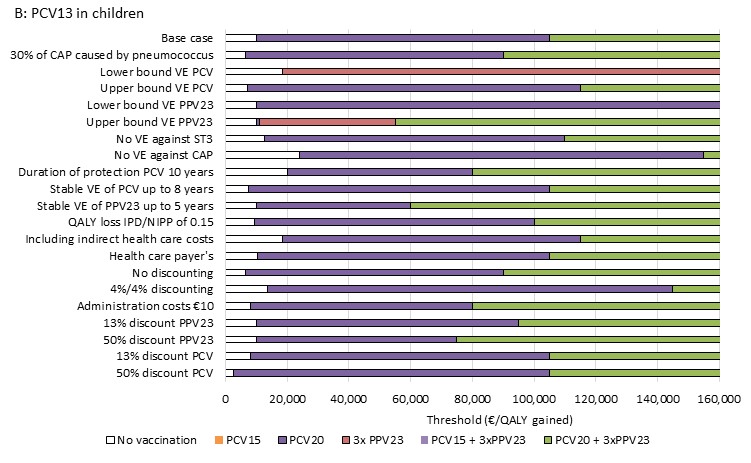


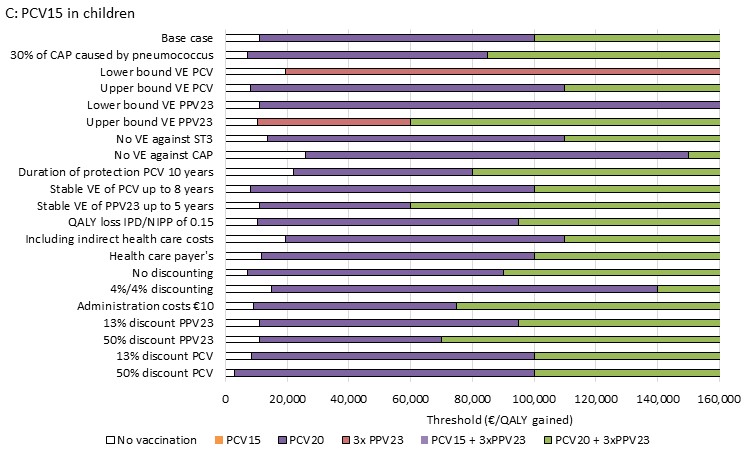


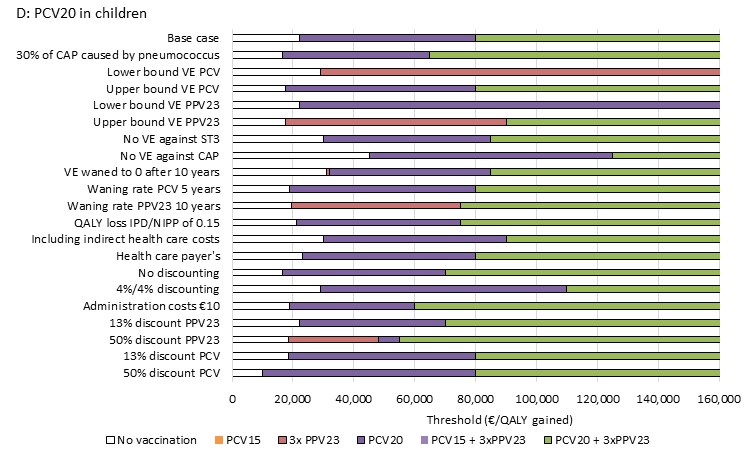


Figure S6: One-way sensitivity analysis of the cost-effectiveness of different pneumococcal vaccination strategies in a 65-years-old cohort (colored bars) while continuing PCV10 in children (panel A), or with a switch to PCV13 (Panel B), PCV15 (Panel C) or PCV20 (Panel D). The bars show the cost-effective strategy across a range of willingness-to-pay thresholds. CAP: Community-acquired pneumonia, ICER: Incremental cost-effectiveness ratio, IPD: Invasive pneumococcal disease, NIPP: Non-invasive pneumococcal pneumonia, PCV: Pneumococcal conjugate vaccine, PPV: pneumococcal polysaccharide vaccine, QALY: Quality-adjusted life year. VE: Vaccine effectiveness, ST: Serotype

## Effect of vaccination age

### Cost-effectiveness compared to no vaccination, PCV10 in children

While the main manuscript and abovementioned results were obtained for a cohort vaccinated at 65 years of age, we also determined the effect of vaccination age at time of vaccination on the ICER (Figure S7). The effect of vaccination age on the impact, number needed to vaccinate, and cost-effectiveness compared to no vaccination is shown for vaccination age 60 years (Table S15), 70 years (Table S16), 75 years (Table S17), 80 years (Table S18) and 85 years (Table S19). The ICERs for different vaccination ages are presented in table S19-S23 for vaccination ages 60-85 years, and are presented in Figure S8. Overall, the ICERs ranked similarly for the different strategies independent of age for the range of 60-80 years. However, the ICER of pneumococcal vaccination largely increased at a vaccination age of 85 years.


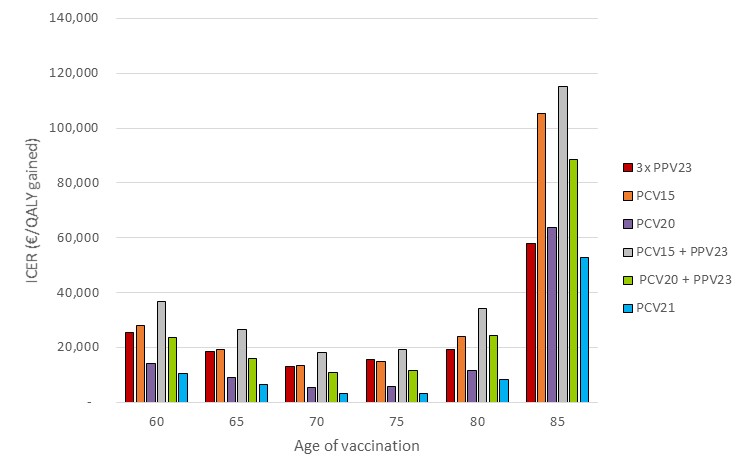


Figure S7: Cost-effectiveness of various vaccination pneumococcal vaccination strategies compared to no vaccination by vaccination age, while PCV10 is continued in children. ICER: Incremental cost-effectiveness ratio, PCV: Pneumococcal conjugate vaccine, PPV: pneumococcal polysaccharide vaccine, QALY: Quality-adjusted life year.

### Clinical impact and cost-effectiveness tables compared to no pneumococcal vaccination, PCV10 in children

Table S15: Clinical impact, number needed to vaccinate and cost-effectiveness of different pneumococcal vaccination strategies in a 60-year-old cohort, while PCV10 is continued in children. The analysis assumed an uptake of 70% in older adults.

| Outcome | No vaccination | Difference to no vaccination | | | | | |
| --- | --- | --- | --- | --- | --- | --- | --- |
|  |  | 3xPPV23 | PCV15 | PCV20 | PCV15+ 3xPPV23 | PCV20+ 3xPPV23 | PCV21 |
| PPV23 administered | - | 514,586 | - | - | 509,776 | 509,785 | - |
| PCV administered | - | - | 178,444 | 178,444 | 178,444 | 178,444 | 178,444 |
| *Clinical outcomes* |  |  |  |  |  |  |  |
| IPD hospitalizations | 1,290 | -263 | -191 | -328 | -348 | -406 | -389 |
| NIPP hospitalizations | 1,633 | -171 | -150 | -259 | -248 | -301 | -307 |
| IPD deaths | 162 | -33 | -21 | -36 | -42 | -48 | -43 |
| NIPP deaths | 180 | -19 | -15 | -25 | -26 | -31 | -30 |
| *Costs* |  |  |  |  |  |  |  |
| PPV23 vaccination | - | 20.22 | - | - | 19.27 | 19.27 | - |
| PCV vaccination | 12.97 | - | 17.08 | 18.55 | 17.08 | 18.55 | 18.55 |
| IPD medical costs | 14.16 | -2.70 | -2.09 | -3.60 | -3.61 | -4.29 | -4.27 |
| NIPP medical costs | 0.14 | -1.49 | -1.35 | -2.32 | -2.18 | -2.67 | -2.75 |
| IPD patient costs | 0.21 | -0.03 | -0.02 | -0.04 | -0.04 | -0.05 | -0.05 |
| NIPP patient costs | 2.44 | -0.02 | -0.02 | -0.03 | -0.03 | -0.04 | -0.04 |
| IPD productivity losses | 2.11 | -0.51 | -0.54 | -0.93 | -0.79 | -1.00 | -1.10 |
| NIPP productivity losses | - | -0.22 | -0.28 | -0.47 | -0.39 | -0.51 | -0.56 |
| *QALY loss* |  |  |  |  |  |  |  |
| IPD hospitalization | 82 | -17 | -13 | -22 | -22 | -26 | -26 |
| NIPP hospitalization | 84 | -9 | -8 | -15 | -13 | -17 | -17 |
| IPD death | 1,880 | -394 | -279 | -481 | -508 | -597 | -570 |
| NIPP death | 1,645 | -179 | -160 | -275 | -256 | -316 | -326 |
| *Total* |  |  |  |  |  |  |  |
| Costs | 32.03 | 15.25 | 12.79 | 11.15 | 29.32 | 29.26 | 9.78 |
| Vaccination | - | 20.22 | 17.08 | 18.55 | 36.36 | 37.82 | 18.55 |
| IPD | 15.55 | -3.23 | -2.65 | -4.57 | -4.43 | -5.34 | -5.42 |
| NIPP | 16.48 | -1.74 | -1.64 | -2.83 | -2.61 | -3.22 | -3.35 |
| QALY loss | 3,691 | -599 | -460 | -792 | -800 | -956 | -939 |
| IPD | 1,962 | -411 | -292 | -503 | -531 | -624 | -596 |
| NIPP | 1,729 | -188 | -168 | -289 | -269 | -332 | -343 |
| *Relative to no vaccination* |  |  |  |  |  |  |  |
| NNV per IPD case avoided |  | 680 | 936 | 544 | 513 | 439 | 459 |
| NNV per NIPP hospitalization avoided |  | 1,046 | 1,188 | 690 | 719 | 592 | 582 |
| NNV per death avoided |  | 3,444 | 4,992 | 2,899 | 2,629 | 2,264 | 2,446 |
| ICER (€/QALY gained) |  | 25,449 | 27,805 | 14,085 | 36,647 | 30,602 | 10,423 |

ICER: Incremental cost-effectiveness ratio, IPD: Invasive pneumococcal disease, NIPP: Non-invasive pneumococcal pneumonia, NNV: Number needed to vaccinate, PCV: Pneumococcal conjugate vaccine, PPV: pneumococcal polysaccharide vaccine, QALY: Quality-adjusted life year

Table S16: Clinical impact, number needed to vaccinate and cost-effectiveness of different pneumococcal vaccination strategies in a 70-year-old cohort, while PCV10 is continued in children. The analysis assumed an uptake of 70% in older adults.

| Outcome | No vaccination | Difference to no vaccination | | | | | |
| --- | --- | --- | --- | --- | --- | --- | --- |
|  |  | 3xPPV23 | PCV15 | PCV20 | PCV15+ 3xPPV23 | PCV20+ 3xPPV23 | PCV21 |
| PPV23 administered | - | 372,049 | - | - | 362,583 | 362,597 | - |
| PCV administered | - | - | 137,480 | 137,480 | 137,480 | 137,480 | 137,480 |
| *Clinical outcomes* |  |  |  |  |  |  |  |
| IPD hospitalizations | 1,356 | -262 | -203 | -349 | -346 | -409 | -414 |
| NIPP hospitalizations | 2,849 | -237 | -232 | -399 | -341 | -424 | -473 |
| IPD deaths | 255 | -49 | -35 | -61 | -63 | -73 | -72 |
| NIPP deaths | 466 | -37 | -35 | -60 | -52 | -64 | -71 |
| *Costs* |  |  |  |  |  |  |  |
| PPV23 vaccination | - | 14.76 | - | - | 13.85 | 13.85 | - |
| PCV vaccination | 17.52 | - | 13.16 | 14.29 | 13.16 | 14.29 | 14.29 |
| IPD medical costs | 24.66 | -3.47 | -2.67 | -4.59 | -4.49 | -5.32 | -5.44 |
| NIPP medical costs | 0.15 | -2.09 | -2.07 | -3.57 | -3.03 | -3.79 | -4.23 |
| IPD patient costs | 0.37 | -0.03 | -0.02 | -0.04 | -0.04 | -0.05 | -0.05 |
| NIPP patient costs | 0.35 | -0.03 | -0.03 | -0.05 | -0.04 | -0.06 | -0.06 |
| IPD productivity losses | 1.11 | -0.07 | -0.08 | -0.14 | -0.11 | -0.15 | -0.16 |
| NIPP productivity losses | - | -0.10 | -0.11 | -0.19 | -0.15 | -0.20 | -0.22 |
| *QALY loss* |  |  |  |  |  |  |  |
| IPD hospitalization | 87 | -17 | -13 | -23 | -22 | -27 | -27 |
| NIPP hospitalization | 150 | -13 | -13 | -23 | -19 | -24 | -27 |
| IPD death | 1,977 | -399 | -319 | -549 | -522 | -627 | -650 |
| NIPP death | 2,856 | -260 | -262 | -452 | -375 | -476 | -535 |
| *Total* |  |  |  |  |  |  |  |
| Costs | 44.16 | 8.97 | 8.18 | 5.71 | 19.14 | 18.59 | 4.12 |
| Vaccination | - | 14.76 | 13.16 | 14.29 | 27.01 | 28.14 | 14.29 |
| IPD | 18.01 | -3.57 | -2.77 | -4.77 | -4.64 | -5.51 | -5.66 |
| NIPP | 26.14 | -2.22 | -2.21 | -3.81 | -3.23 | -4.04 | -4.51 |
| QALY loss | 5,070 | -689 | -607 | -1,046 | -939 | -1,155 | -1,240 |
| IPD | 2,064 | -416 | -332 | -572 | -544 | -654 | -678 |
| NIPP | 3,006 | -273 | -275 | -474 | -394 | -500 | -562 |
| *Relative to no vaccination* |  |  |  |  |  |  |  |
| NNV per IPD case avoided |  | 524 | 678 | 394 | 397 | 336 | 332 |
| NNV per NIPP hospitalization avoided |  | 581 | 593 | 345 | 403 | 324 | 291 |
| NNV per death avoided |  | 1,598 | 1,959 | 1,138 | 1,196 | 999 | 960 |
| ICER (€/QALY gained) |  | 13,012 | 13,462 | 5,459 | 20,393 | 16,106 | 3,323 |

ICER: Incremental cost-effectiveness ratio, IPD: Invasive pneumococcal disease, NIPP: Non-invasive pneumococcal pneumonia, NNV: Number needed to vaccinate, PCV: Pneumococcal conjugate vaccine, PPV: pneumococcal polysaccharide vaccine, QALY: Quality-adjusted life year

Table S17: Clinical impact, number needed to vaccinate and cost-effectiveness of different pneumococcal vaccination strategies in a 75-year-old cohort, while PCV10 is continued in children. The analysis assumed an uptake of 70% in older adults.

| Outcome | No vaccination | Difference to no vaccination | | | | | |
| --- | --- | --- | --- | --- | --- | --- | --- |
|  |  | 3xPPV23 | PCV15 | PCV20 | PCV15+ 3xPPV23 | PCV20+ 3xPPV23 | PCV21 |
| PPV23 administered | - | 310,369 | - | - | 296,819 | 296,831 | - |
| PCV administered | - | - | 123,391 | 123,391 | 123,391 | 123,391 | 123,391 |
| *Clinical outcomes* |  |  |  |  |  |  |  |
| IPD hospitalizations | 1,264 | -219 | -177 | -304 | -257 | -320 | -360 |
| NIPP hospitalizations | 3,282 | -164 | -218 | -375 | -292 | -390 | -445 |
| IPD deaths | 264 | -45 | -35 | -60 | -51 | -63 | -71 |
| NIPP deaths | 591 | -27 | -37 | -64 | -50 | -67 | -76 |
| *Costs* |  |  |  |  |  |  |  |
| PPV23 vaccination | - | 12.45 | - | - | 11.49 | 11.49 | - |
| PCV vaccination | 16.32 | - | 11.81 | 12.83 | 11.81 | 12.83 | 12.83 |
| IPD medical costs | 26.15 | -3.05 | -2.67 | -4.60 | -3.92 | -4.85 | -5.45 |
| NIPP medical costs | 0.14 | -1.51 | -1.88 | -3.24 | -2.54 | -3.37 | -3.84 |
| IPD patient costs | 0.43 | -0.03 | -0.02 | -0.04 | -0.03 | -0.04 | -0.04 |
| NIPP patient costs | - | -0.02 | -0.03 | -0.05 | -0.04 | -0.05 | -0.06 |
| IPD productivity losses | 0.97 | - | - | - | - | - | - |
| NIPP productivity losses | - | -0.05 | -0.07 | -0.11 | -0.09 | -0.12 | -0.14 |
| *QALY loss* |  |  |  |  |  |  |  |
| IPD hospitalization | 81 | -14 | -12 | -20 | -17 | -21 | -24 |
| NIPP hospitalization | 178 | -10 | -13 | -22 | -17 | -23 | -26 |
| IPD death | 1,576 | -291 | -239 | -412 | -349 | -434 | -488 |
| NIPP death | 2,910 | -185 | -219 | -377 | -296 | -393 | -447 |
| *Total* |  |  |  |  |  |  |  |
| Costs | 44.02 | 7.80 | 7.15 | 4.79 | 16.67 | 15.88 | 3.30 |
| Vaccination | - | 12.45 | 11.81 | 12.83 | 23.30 | 24.31 | 12.83 |
| IPD | 16.46 | -3.07 | -2.69 | -4.63 | -3.95 | -4.89 | -5.49 |
| NIPP | 27.56 | -1.58 | -1.98 | -3.40 | -2.67 | -3.54 | -4.03 |
| QALY loss | 4,745 | -501 | -482 | -831 | -678 | -870 | -985 |
| IPD | 1,657 | -305 | -251 | -432 | -366 | -455 | -512 |
| NIPP | 3,088 | -195 | -232 | -399 | -313 | -415 | -473 |
| *Relative to no vaccination* |  |  |  |  |  |  |  |
| NNV per IPD case avoided |  | 565 | 699 | 406 | 480 | 385 | 342 |
| NNV per NIPP hospitalization avoided |  | 751 | 566 | 329 | 423 | 316 | 277 |
| NNV per death avoided |  | 1,712 | 1,711 | 994 | 1,229 | 950 | 838 |
| ICER (€/QALY gained) |  | 15,573 | 14,814 | 5,770 | 24,576 | 18,250 | 3,355 |

ICER: Incremental cost-effectiveness ratio, IPD: Invasive pneumococcal disease, NIPP: Non-invasive pneumococcal pneumonia, NNV: Number needed to vaccinate, PCV: Pneumococcal conjugate vaccine, PPV: pneumococcal polysaccharide vaccine, QALY: Quality-adjusted life year

Table S18: Clinical impact, number needed to vaccinate and cost-effectiveness of different pneumococcal vaccination strategies in a 80-year-old cohort, while PCV10 is continued in children. The analysis assumed an uptake of 70% in older adults. We assumed no PPV23 vaccination above the age of 86 years (maximum of 2x PPV23).

| Outcome | No vaccination | Difference to no vaccination | | | | | |
| --- | --- | --- | --- | --- | --- | --- | --- |
|  |  | 3xPPV23 | PCV15 | PCV20 | PCV15+ 3xPPV23 | PCV20+ 3xPPV23 | PCV21 |
| PPV23 administered | - | 156,561 | - | - | 147,643 | 147,649 | - |
| PCV administered | - | - | 89,009 | 89,009 | 89,009 | 89,009 | 89,009 |
| *Clinical outcomes* |  |  |  |  |  |  |  |
| IPD hospitalizations | 855 | -110 | -108 | -186 | -140 | -192 | -220 |
| NIPP hospitalizations | 2,509 | -63 | -110 | -190 | -136 | -195 | -225 |
| IPD deaths | 213 | -26 | -26 | -44 | -33 | -46 | -53 |
| NIPP deaths | 532 | -12 | -22 | -39 | -28 | -40 | -46 |
| *Costs* |  |  |  |  |  |  |  |
| PPV23 vaccination | - | 6.78 | - | - | 6.16 | 6.16 | - |
| PCV vaccination | 9.64 | - | 8.52 | 9.25 | 8.52 | 9.25 | 9.25 |
| IPD medical costs | 18.85 | -1.51 | -1.41 | -2.43 | -1.94 | -2.54 | -2.88 |
| NIPP medical costs | 0.10 | -0.60 | -0.88 | -1.52 | -1.12 | -1.57 | -1.81 |
| IPD patient costs | 0.33 | -0.01 | -0.01 | -0.02 | -0.02 | -0.02 | -0.03 |
| NIPP patient costs | - | -0.01 | -0.01 | -0.03 | -0.02 | -0.03 | -0.03 |
| IPD productivity losses | 0.76 | - | - | - | - | - | - |
| NIPP productivity losses | - | -0.02 | -0.03 | -0.06 | -0.04 | -0.06 | -0.07 |
| *QALY loss* |  |  |  |  |  |  |  |
| IPD hospitalization | 56 | -7 | -7 | -12 | -9 | -13 | -15 |
| NIPP hospitalization | 143 | -4 | -7 | -11 | -8 | -12 | -14 |
| IPD death | 1,001 | -148 | -138 | -237 | -183 | -246 | -281 |
| NIPP death | 2,161 | -80 | -107 | -184 | -136 | -190 | -218 |
| *Total* |  |  |  |  |  |  |  |
| Costs | 29.68 | 4.63 | 6.16 | 5.19 | 11.54 | 11.19 | 4.44 |
| Vaccination | - | 6.78 | 8.52 | 9.25 | 14.68 | 15.42 | 9.25 |
| IPD | 9.74 | -1.52 | -1.43 | -2.46 | -1.96 | -2.56 | -2.91 |
| NIPP | 19.94 | -0.63 | -0.93 | -1.61 | -1.18 | -1.66 | -1.91 |
| QALY loss | 3,360 | -240 | -258 | -444 | -336 | -460 | -527 |
| IPD | 1,057 | -155 | -145 | -249 | -192 | -259 | -296 |
| NIPP | 2,304 | -84 | -113 | -195 | -144 | -201 | -231 |
| *Relative to no vaccination* |  |  |  |  |  |  |  |
| NNV per IPD case avoided |  | 809 | 824 | 479 | 638 | 463 | 404 |
| NNV per NIPP hospitalization avoided |  | 1,412 | 807 | 468 | 653 | 456 | 395 |
| NNV per death avoided |  | 2,339 | 1,845 | 1,072 | 1,467 | 1,040 | 904 |
| ICER (€/QALY gained) |  | 19,333 | 23,872 | 11,676 | 34,319 | 24,322 | 8,421 |

ICER: Incremental cost-effectiveness ratio, IPD: Invasive pneumococcal disease, NIPP: Non-invasive pneumococcal pneumonia, NNV: Number needed to vaccinate, PCV: Pneumococcal conjugate vaccine, PPV: pneumococcal polysaccharide vaccine, QALY: Quality-adjusted life year

Table S19: Clinical impact, number needed to vaccinate and cost-effectiveness of different pneumococcal vaccination strategies in a 85-year-old cohort, while PCV10 is continued in children. The analysis assumed an uptake of 70% in older adults. We assumed no PPV23 vaccination above the age of 86 years (maximum of 1x PPV23).

| Outcome | No vaccination | Difference to no vaccination | | | | | |
| --- | --- | --- | --- | --- | --- | --- | --- |
|  |  | 3xPPV23 | PCV15 | PCV20 | PCV15+ 3xPPV23 | PCV20+ 3xPPV23 | PCV21 |
| PPV23 administered | - | 55,006 | - | - | 50,768 | 50,768 | - |
| PCV administered | - | - | 55,006 | 55,006 | 55,006 | 55,006 | 55,006 |
| *Clinical outcomes* |  |  |  |  |  |  |  |
| IPD hospitalizations | 427 | -34 | -38 | -66 | -38 | -66 | -78 |
| NIPP hospitalizations | 1,362 | 0 | -7 | -12 | -7 | -12 | -14 |
| IPD deaths | 114 | -8 | -10 | -17 | -10 | -17 | -20 |
| NIPP deaths | 308 | 0 | -1 | -3 | -1 | -3 | -3 |
| *Costs* |  |  |  |  |  |  |  |
| PPV23 vaccination | - | 2.58 | - | - | 2.29 | 2.29 | - |
| PCV vaccination | 3.14 | - | 5.27 | 5.72 | 5.27 | 5.72 | 5.72 |
| IPD medical costs | 9.07 | -0.28 | -0.29 | -0.50 | -0.29 | -0.50 | -0.59 |
| NIPP medical costs | 0.05 | 0.00 | -0.05 | -0.08 | -0.05 | -0.08 | -0.09 |
| IPD patient costs | 0.18 | -0.00 | -0.00 | -0.01 | -0.00 | -0.01 | -0.01 |
| NIPP patient costs | - | 0.00 | -0.00 | -0.00 | -0.00 | -0.00 | -0.00 |
| IPD productivity losses | 0.42 | - | - | - | - | - | - |
| NIPP productivity losses | - | 0.00 | -0.00 | -0.00 | -0.00 | -0.00 | -0.00 |
| *QALY loss* |  |  |  |  |  |  |  |
| IPD hospitalization | 28 | -2 | -3 | -4 | -3 | -4 | -5 |
| NIPP hospitalization | 82 | 0 | -0 | -1 | -0 | -1 | -1 |
| IPD death | 408 | -37 | -38 | -66 | -38 | -66 | -78 |
| NIPP death | 1,002 | 0 | -5 | -9 | -5 | -9 | -11 |
| *Total* |  |  |  |  |  |  |  |
| Costs | 12.87 | 2.30 | 4.92 | 5.12 | 7.21 | 7.41 | 5.01 |
| Vaccination | - | 2.58 | 5.27 | 5.72 | 7.56 | 8.01 | 5.72 |
| IPD | 3.19 | -0.28 | -0.30 | -0.51 | -0.30 | -0.51 | -0.60 |
| NIPP | 9.67 | 0.00 | -0.05 | -0.09 | -0.05 | -0.09 | -0.10 |
| QALY loss | 1,521 | -40 | -47 | -80 | -47 | -80 | -95 |
| IPD | 437 | -40 | -41 | -70 | -41 | -70 | -83 |
| NIPP | 1,084 | 0 | -6 | -10 | -6 | -10 | -12 |
| *Relative to no vaccination* |  |  |  |  |  |  |  |
| NNV per IPD case avoided |  | 1,607 | 1,433 | 832 | 1,433 | 832 | 702 |
| NNV per NIPP hospitalization avoided |  | -459,536 | 8,021 | 4,658 | 8,021 | 4,658 | 3,930 |
| NNV per death avoided |  | 6,921 | 4,838 | 2,809 | 4,838 | 2,809 | 2,370 |
| ICER (€/QALY gained) |  | 57,957 | 105,432 | 63,749 | 154,535 | 92,265 | 52,623 |

ICER: Incremental cost-effectiveness ratio, IPD: Invasive pneumococcal disease, NIPP: Non-invasive pneumococcal pneumonia, NNV: Number needed to vaccinate, PCV: Pneumococcal conjugate vaccine, PPV: pneumococcal polysaccharide vaccine, QALY: Quality-adjusted life year

### Incremental cost-effectiveness tables, PCV10 in children

Table S20: Incremental cost-effectiveness ratio of different vaccination strategies in a 60-year-olds cohort, while remaining PC10 in children. The analysis assumed an uptake of 70% in older adults.

| Schedule | Total QALY loss | Total costs (€, millions) | Comparator | Incremental QALYs | Incremental costs (€, millions) | ICER (€/QALY gained) |
| --- | --- | --- | --- | --- | --- | --- |
| No vaccination | 3,691 | 32.03 |  |  |  |  |
| PCV15 | 3,231 | 44.82 | No vaccination | 460 | 12.79 | Dominated |
| 3xPPV23 | 3,092 | 47.28 | No vaccination | 599 | 15.25 | Dominated |
| PCV20 | 2,899 | 43.18 | No vaccination | 792 | 11.15 | 14,085 |
| PCV15+3xPPV23 | 2,891 | 61.35 | PCV20 | 8 | 18 | Dominated |
| *PCV21** | *2,752* | *41.81* | *No vaccination* | *939* | *9.78* | *10,423* |
| PCV20+3xPPV23 | 2,735 | 61.29 | PCV20 | 164 | 18 | 110,264 |

* As PCV21 is currently under development, we assumed its vaccine price to be equal to PCV20. Therefore, this strategy was excluded from the incremental comparison and only indicatively compared with no vaccination. ICER: Incremental cost-effectiveness ratio, PCV: Pneumococcal conjugate vaccine, PPV: pneumococcal polysaccharide vaccine, QALY: Quality-adjusted life year.

Table S21: Incremental cost-effectiveness ratio of different vaccination strategies in a 70-year-olds cohort, while remaining PC10 in children. The analysis assumed an uptake of 70% in older adults.

| Schedule | Total QALY loss | Total costs (€, millions) | Comparator | Incremental QALYs | Incremental costs (€, millions) | ICER (€/QALY gained) |
| --- | --- | --- | --- | --- | --- | --- |
| No vaccination | 5,070 | 44.16 |  |  |  |  |
| PCV15 | 4,462 | 52.33 | No vaccination | 607 | 8.18 | Dominated |
| 3xPPV23 | 4,380 | 53.12 | No vaccination | 689 | 8.97 | Dominated |
| PCV15+3xPPV23 | 4,131 | 63.29 | No vaccination | 939 | 19.14 | Dominated |
| PCV20 | 4,024 | 49.87 | No vaccination | 1,046 | 5.71 | 5,459 |
| PCV20+3xPPV23 | 3,915 | 62.75 | PCV20 | 108 | 12.88 | 118,765 |
| *PCV21** | *3,830* | *48.28* | *No vaccination* | *1,240* | *4.12* | *3,323* |

* As PCV21 is currently under development, we assumed its vaccine price to be equal to PCV20. Therefore, this strategy was excluded from the incremental comparison and only indicatively compared with no vaccination. ICER: Incremental cost-effectiveness ratio, PCV: Pneumococcal conjugate vaccine, PPV: pneumococcal polysaccharide vaccine, QALY: Quality-adjusted life year.

Table S22: Incremental cost-effectiveness ratio of different vaccination strategies in a 75-year-olds cohort, while remaining PC10 in children. The analysis assumed an uptake of 70% in older adults.

| Schedule | Total QALY loss | Total costs (€, millions) | Comparator | Incremental QALYs | Incremental costs (€, millions) | ICER (€/QALY gained) |
| --- | --- | --- | --- | --- | --- | --- |
| No vaccination | 4,745 | 44.02 |  |  |  |  |
| PCV15 | 4,263 | 51.17 | No vaccination | 482 | 7.15 | Dominated |
| 3x PPV23 | 4,244 | 51.82 | No vaccination | 501 | 7.80 | Dominated |
| PCV15+3xPPV23 | 4,042 | 60.55 | No vaccination | 703 | 16.53 | Dominated |
| PCV20 | 3,914 | 48.81 | No vaccination | 831 | 4.79 | 5,770 |
| PCV20+3xPPV23 | 3,859 | 59.81 | PCV20 | 55 | 11.00 | 198,264 |
| *PCV21** | *3,760* | *47.32* | *No vaccination* | *985* | *3.30* | *3,355* |

* As PCV21 is currently under development, we assumed its vaccine price to be equal to PCV20. Therefore, this strategy was excluded from the incremental comparison and only indicatively compared with no vaccination. ICER: Incremental cost-effectiveness ratio, PCV: Pneumococcal conjugate vaccine, PPV: pneumococcal polysaccharide vaccine, QALY: Quality-adjusted life year.

Table S23: Incremental cost-effectiveness ratio of different vaccination strategies in a 80-year-olds cohort, while remaining PC10 in children. The analysis assumed an uptake of 70% in older adults.

| Schedule | Total QALY loss | Total costs (€, millions) | Comparator | Incremental QALYs | Incremental costs (€, millions) | ICER (€/QALY gained) |
| --- | --- | --- | --- | --- | --- | --- |
| No vaccination | 3,360 | 29.68 |  |  |  |  |
| 2xPPV23^$^ | 3,121 | 34.31 | No vaccination | 240 | 4.63 | Dominated |
| PCV15 | 3,102 | 35.84 | No vaccination | 258 | 6.16 | Dominated |
| PCV15+2xPPV23^$^ | 3,007 | 41.11 | No vaccination | 353 | 11.43 | Dominated |
| PCV20 | 2,916 | 34.87 | No vaccination | 444 | 5.19 | 11,676 |
| PCV20+2xPPV23^$^ | 2,897 | 40.85 | PCV20 | 19 | 5.98 | 309,958 |
| *PCV21** | *2,834* | *34.11* | *No vaccination* | *527* | *4.44* | *8,421* |

* As PCV21 is currently under development, we assumed its vaccine price to be equal to PCV20. Therefore, this strategy was excluded from the incremental comparison and only indicatively compared with no vaccination. ICER: Incremental cost-effectiveness ratio, PCV: Pneumococcal conjugate vaccine, PPV: pneumococcal polysaccharide vaccine, QALY: Quality-adjusted life year.
^$^We assumed no PPV23 vaccination above the age of 86 years

Table S24: Incremental cost-effectiveness ratio of different vaccination strategies in a 85-year-olds cohort, while remaining PC10 in children. The analysis assumed an uptake of 70% in older adults.

| Schedule | Total QALY loss | Total costs (€, millions) | Comparator | Incremental QALYs | Incremental costs (€, millions) | ICER (€/QALY gained) |
| --- | --- | --- | --- | --- | --- | --- |
| No vaccination | 1,521 | 12.87 |  |  |  |  |
| PPV23^$^ | 1,481 | 15.17 | No vaccination | 40 | 2.30 | 57,957 |
| PCV15 | 1,474 | 17.79 | PPV23 | 7 | 2.62 | Dominated |
| PCV15+PPV23^$^ | 1,459 | 19.97 | PPV23 | 22 | 4.80 | Dominated |
| PCV20 | 1,441 | 17.99 | PPV23 | 41 | 2.82 | 69,392 |
| PCV20+PPV23^$^ | 1,438 | 20.26 | PCV20 | 3 | 2.27 | 746,328 |
| *PCV21** | *1,426* | *17.88* | *No vaccination* | *95* | *5.01* | *52,623* |

* As PCV21 is currently under development, we assumed its vaccine price to be equal to PCV20. Therefore, this strategy was excluded from the incremental comparison and only indicatively compared with no vaccination. ICER: Incremental cost-effectiveness ratio, PCV: Pneumococcal conjugate vaccine, PPV: pneumococcal polysaccharide vaccine, QALY: Quality-adjusted life year.
^$^ We assumed no PPV23 vaccination above the age of 86 years

### Cost-effectiveness compared to no vaccination, different childhood vaccine

Figure S8 shows the cost-effectiveness of different vaccination strategies compared to no vaccination in older adults per vaccination age (panels), while varying the childhood vaccine (bars).


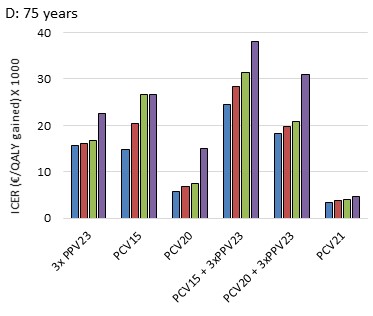


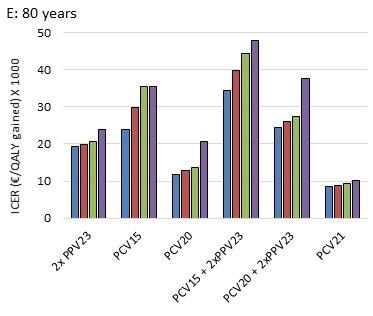

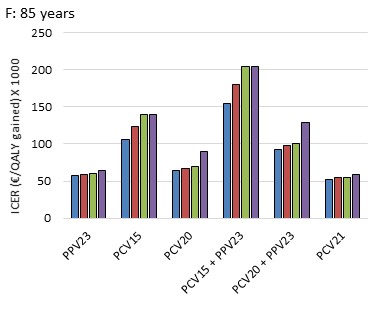


Figure S8: Cost-effectiveness of different pneumococcal vaccination strategies in older adults for different vaccination ages in the age-range 60 to 85 years (Panel A-F), while varying the childhood vaccine between PCV10 (blue bars), PCV13 (red bars), PCV15 (green bars), and PCV20 (purple bars). The results present the ICERs compared to no vaccination. We assumed no PPV23 vaccination above the age of 86 years. ICER: Incremental cost-effectiveness ratio, PCV: Pneumococcal conjugate vaccine, PPV: pneumococcal polysaccharide vaccine, QALY: Quality-adjusted life year.
